# Chitosan-confined selenium–silver nanohybrids enable redox-modulated mitochondrial apoptosis and survival improvement in experimental hepatocellular carcinoma

**DOI:** 10.1039/d6ra01968d

**Published:** 2026-06-05

**Authors:** Youssef M. Hassan, Doaa A. Elawady, Ahmed Wanas, Hala El-Tantawi, Dalia M. El-Husseini

**Affiliations:** a Department of Zoology, Faculty of Science, Ain Shams University Abbassia 11566 Cairo Egypt youssefmuhammedbio@gmail.com; b Department of Biochemistry, Faculty of Science, Ain Shams University Abbassia 11566 Cairo Egypt; c Nanomaterial Research and Synthesis Unit, Animal Health Research Institute, Agricultural Research Center (ARC) Dokki Giza Egypt

## Abstract

Polymer-confined multi-metal nanostructures offer a strategy to modulate tumor redox balance with improved selectivity. Here, we engineered chitosan-stabilized selenium–silver (Se–Ag) nanohybrids as redox-regulating agents for hepatocellular carcinoma (HCC). The nanocomposite was synthesized *via* green routes, combining phytochemical reduction of silver nanoparticles from *Allium cepa* extract with ascorbic acid-reduced selenium nanoparticles, followed by confinement within a low-molecular-weight chitosan matrix. Transmission electron microscopy revealed discrete metallic nanodomains (45–205 nm) embedded in a continuous polymer scaffold, while inductively coupled plasma optical emission spectroscopy confirmed reproducible elemental loading (Se: 50.0 ± 2.5 mg L^−1^; Ag: 6.0 ± 0.3 mg L^−1^) and buffer stability. In a diethylnitrosamine-induced murine HCC model, treatment significantly improved survival, reduced tumor burden, and preserved hepatic architecture. Preferential hepatic accumulation (liver : kidney ratio 5.8 : 1) was observed. Mechanistically, the nanohybrids restored redox balance, reduced lipid peroxidation, enhanced glutathione and superoxide dismutase activity, and activated intrinsic mitochondrial apoptosis *via* the p53–BAX–caspase-9 pathway. Apoptosis was selectively localized to tumor tissue. These results establish a structure–function relationship in which chitosan-mediated confinement regulates metal release, dual-metal integration amplifies tumor-specific oxidative stress, and hepatic targeting enhances therapeutic index, supporting Se–Ag nanohybrids as a materials-driven platform for redox-based liver cancer therapy.

## Abstract 2

Polymer-confined multi-metal nanostructures offer a strategy to modulate tumor redox balance with improved selectivity.

## Graphical abstract

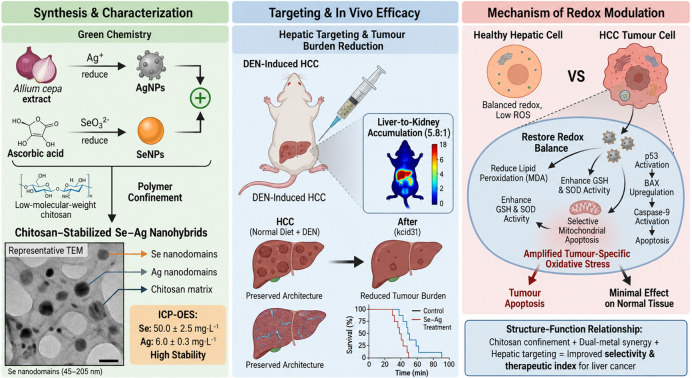

## 1 Introduction

Hepatocellular carcinoma (HCC) represents the predominant primary liver malignancy and remains one of the leading causes of cancer-related mortality globally, with a steadily increasing incidence driven by chronic viral hepatitis, metabolic syndrome, and non-alcoholic fatty liver disease.^1,2^ Despite advances in surgical resection, locoregional therapies, and targeted agents such as multikinase inhibitors and immune checkpoint blockade, overall survival remains limited due to late-stage diagnosis, high recurrence rates, therapeutic resistance, and tumor heterogeneity.^3^ The complex molecular landscape of HCC, characterized by dysregulated cell-cycle signaling, chronic inflammation, angiogenesis, and metabolic reprogramming, underscores the need for innovative therapeutic strategies that selectively target tumor-specific vulnerabilities.^4^

Among these vulnerabilities, oxidative stress imbalance and mitochondrial dysfunction play central roles in hepatocarcinogenesis.^5^ HCC cells exhibit elevated basal reactive oxygen species levels due to oncogenic signaling and metabolic reprogramming; however, they simultaneously upregulate antioxidant defenses to maintain survival within a pro-oxidant microenvironment.^6^ This redox adaptation creates a therapeutic window in which further oxidative perturbation can selectively trigger mitochondrial membrane depolarization, cytochrome c release, caspase activation, and intrinsic apoptosis in malignant hepatocytes while sparing normal tissue.^7^ Consequently, controlled redox modulation has emerged as a promising strategy for tumor-selective intervention.^8^

Nanotechnology offers unique opportunities to exploit this redox vulnerability through precise control of particle size, surface chemistry, composition, and biodistribution.^9,10^ Nanoscale systems can enhance tumor accumulation *via* enhanced permeability and retention effects and modulate intracellular trafficking, thereby improving therapeutic selectivity. Redox-active metallic nanoparticles have attracted particular attention due to their intrinsic capacity to influence oxidative signaling pathways. Selenium nanoparticles (SeNPs) demonstrate dual redox-regulating behavior: at physiological levels they support antioxidant defense, whereas at higher localized concentrations they promote pro-apoptotic signaling with improved bioavailability and reduced systemic toxicity compared with conventional selenium salts.^11,12^

Silver nanoparticles (AgNPs), in contrast, are potent generators of reactive oxygen species and can induce mitochondrial dysfunction, DNA damage, and apoptosis in malignant cells.^13,14^ However, uncontrolled silver ion release may result in nonspecific cytotoxicity, necessitating structural stabilization strategies to achieve therapeutic precision. Rational integration of selenium and silver within a confined nanoscale architecture may therefore enable balanced redox amplification—combining selenium-mediated regulatory effects with silver-driven oxidative stress induction—to achieve tumor-selective cytotoxicity.

Polymeric stabilization represents a critical design parameter in such hybrid systems. Chitosan, a biodegradable cationic polysaccharide derived from chitin, offers multiple advantages including biocompatibility, functional amino groups for metal coordination, enhanced colloidal stability, and favorable interactions with negatively charged cellular membranes.^15^ Moreover, chitosan based nanostructures have demonstrated improved hepatic retention and cellular uptake, attributes particularly relevant for liver-targeted therapy.^16^

Despite these theoretical advantages, the *in vivo* therapeutic potential and mechanistic impact of chitosan-mediated selenium–silver nanohybrids in hepatocellular carcinoma remain insufficiently characterized. In particular, comprehensive integration of physicochemical validation, pharmacokinetic modeling, survival analysis, and molecular apoptosis profiling is lacking.

From a cancer biology perspective, HCC cells are distinguished by several exploitable vulnerabilities: constitutively elevated reactive oxygen species arising from oncogenic signaling, dysregulated mitochondrial membrane potential, p53 pathway aberrations, and a characteristically high cellular turnover rate reflected by elevated Ki-67 indices.^1,2^ These features collectively narrow the therapeutic window for redox-based interventions, as tumor cells operate near the upper limit of oxidative stress tolerance while normal hepatocytes maintain robust antioxidant reserves. Rational exploitation of this differential redox buffering capacity therefore represents a biologically grounded strategy for selective tumor elimination.^7,8^ Recent advances in HCC nanotherapy (2022–2025) have explored diverse nanoplatforms including lipid-polymer hybrid carriers, metal–organic frameworks, and stimuli-responsive nanoparticles for targeted HCC intervention.^34–36^ Despite progress, the majority of these systems rely on single-metal or organic drug formulations that lack the dual-metal redox synergism and simultaneous hepatic targeting afforded by bimetallic polymer-confined systems. Critically, few studies have validated *in vivo* apoptotic signaling through combined immunohistochemical and survival endpoints, and none have integrated physiologically based pharmacokinetic modeling with molecular pathway analysis in the DEN-induced HCC model. The present study was therefore designed to address these specific gaps: (i) integration of dualmetal redox synergy within a single chitosan scaffold, (ii) systematic evaluation of tumor-stagedependent therapeutic response, (iii) mechanistic validation of p53-BAX-caspase-9 apoptotic signaling, and (iv) pharmacokinetic–pharmacodynamic correlation to support rational nanomedicine design.

In the present study, we engineered green-synthesized selenium–silver nanohybrids confined within a chitosan matrix and systematically evaluated their therapeutic performance in a diethylnitrosamine-induced murine model of hepatocellular carcinoma. We combined transmission electron microscopy, dynamic light scattering, zeta potential analysis, and elemental quantification with *in vivo* survival studies, histopathology, immunohistochemistry, oxidative stress biomarker assessment, and physiologically based pharmacokinetic modeling. Our results demonstrate that chitosan-stabilized selenium–silver nanohybrids induce intrinsic mitochondrial apoptosis through p53-associated signaling pathways and significantly improve disease-associated parameters in experimental hepatocellular carcinoma, supporting their development as a materials-driven redox-modulating therapeutic platform.

## 2 Materials and methods

### 2.1 Materials

#### 2.1.1 Chemicals and Reagents

Sodium selenite (Na_2_SeO_3_, ≥99%), silver nitrate (AgNO_3_, ≥99%), diethylnitrosamine, and carbon tetrachloride were obtained from Sigma-Aldrich and Merck and used without further purification. Ascorbic acid, sodium citrate, and glacial acetic acid were of analytical grade. Low-molecular-weight chitosan (degree of deacetylation ≈ 90%) was purchased from Acros Organics. Phosphate-buffered saline (PBS, pH 7.4) and sterile physiological saline were prepared under aseptic conditions. Ultrapure water (18.2 MΩ cm) was used for all preparations.

Commercial assay kits for alanine aminotransferase (ALT), aspartate aminotransferase (AST), alkaline phosphatase (ALP), thiobarbituric acid reactive substances/malondialdehyde (TBARS/MDA), reduced glutathione (GSH), and superoxide dismutase (SOD) were obtained from BioVision (USA) and used according to manufacturer protocols.

Fresh red onion (*Allium cepa*) bulbs were procured from a certified organic supplier and used immediately for plant-mediated silver nanoparticle synthesis^15,17^

### 2.2 Green synthesis and fabrication of Se–Ag/chitosan nanohybrids

#### 2.2.1 Preparation of *Allium cepa* extract

Fresh red onion bulbs (50 g) were washed thoroughly with deionized water, finely chopped, and boiled in 100 mL ultrapure water for 15 min. The extract was filtered through Whatman No. 1 paper to remove solid residues and stored at 4 °C for no longer than 24 h prior to use. The extract contains phenolics, flavonoids, and organosulfur compounds that act as reducing and capping agents during nanoparticle formation.^30,31^

#### 2.2.2 Biosynthesis of silver nanoparticles

Silver nanoparticles were synthesized *via* phytochemical reduction. Briefly, 50 mL of 1 mM AgNO_3_ solution was mixed with 10 mL of freshly prepared *Allium cepa* extract under magnetic stirring at 65 ± 5 °C for 2 h. Reduction of Ag^+^ to metallic Ag^0^ was confirmed by the appearance of a brown coloration and a surface plasmon resonance peak at approximately 420 nm, as determined by ultraviolet-visible spectroscopy. The nanoparticles were collected by centrifugation at 12 000 rpm for 15 min, washed three times with ultrapure water, and redispersed in 1% (w/v) chitosan dissolved in 1% (v/v) acetic acid to ensure polymer-mediated stabilization and prevent aggregation.^30–34^

#### 2.2.3 Biosynthesis of selenium nanoparticles

Selenium nanoparticles were prepared by controlled chemical reduction of selenite. A 50 mL solution of 1 mM Na_2_SeO_3_ was prepared in 1% (w/v) chitosan (pH adjusted to 4.5). Ascorbic acid (2 mM) was added dropwise under continuous stirring at room temperature. Formation of elemental Se^0^ was indicated by the development of a characteristic orange-red color.

The suspension was centrifuged at 12 000 rpm for 15 min, washed with ultrapure water, and redispersed in chitosan solution to maintain colloidal stability and surface functionality.^18–20^

#### 2.2.4 Fabrication of Se–Ag/chitosan hybrid nanocomposite

Equal volumes of stabilized AgNP and SeNP suspensions were combined under continuous magnetic stirring to yield final metal concentrations of 50 mg L^−1^ (Se) and 6 mg L^−1^ (Ag). Sodium citrate (0.1% w/v) was added to promote mild ionic crosslinking within the chitosan network and enhance nanodomain confinement.

The mixture was stirred for 24 h at room temperature to ensure homogeneous integration of redox-active nanodomains. The resulting Se–Ag/chitosan nanohybrid was collected by centrifugation, washed with PBS to remove loosely associated species, lyophilized, and stored at 4 °C in the dark until further use.^21–24^

**Fig. 1:**
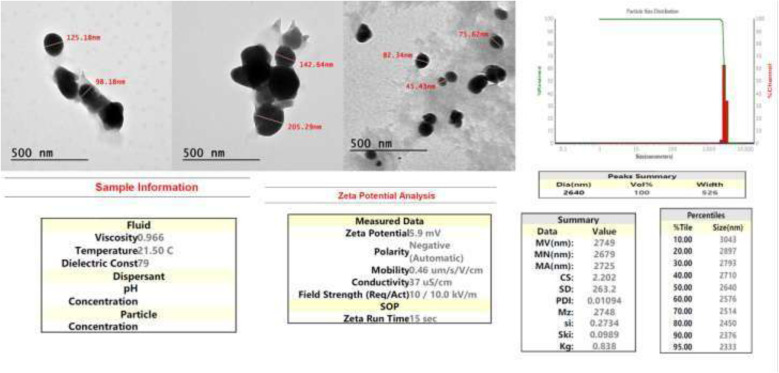
Physicochemical characterization of the Se–Ag/chitosan hybrid nanocomposite.

This fabrication strategy enables nanoscale immobilization of selenium and silver domains within a biodegradable polymer scaffold, minimizing aggregation, modulating metal ion release, and preserving colloidal stability for *in vivo* application (Fig. 1).

Transmission electron microscopy (TEM) images show selenium and silver nanodomains embedded within the chitosan matrix, where discrete primary particles with diameters ranging from ∼45 to 205 nm confirm successful nanoscale formation and spatial confinement within the polymer scaffold (scale bar = 500 nm). The homogeneous nanodomain dispersion indicates effective macromolecular stabilization and prevention of uncontrolled aggregation. Dynamic light scattering (DLS) analysis reveals a mean hydrodynamic diameter (*Z*_avg) of 2749 nm with a very low polydispersity index (PDI = 0.01094). The substantially larger hydrodynamic size relative to TEM-derived core diameters reflects the formation of hydrated chitosan-mediated microclusters and polymer network expansion in aqueous dispersion, rather than metallic core growth. Zeta potential measurement (−5.9 mV) indicates a near-neutral surface charge following chitosan–metal coordination and citrate-mediated crosslinking, suggesting that dispersion stability is predominantly governed by steric stabilization and polymer network confinement rather than electrostatic repulsion alone. DLS-derived distribution parameters, including mean number diameter (MN = 2679 nm), intensity-weighted diameter (Mz = 2748 nm), and standard deviation (SD = 263.2 nm), further support the presence of hydrated polymeric microassemblies with a narrow size distribution in aqueous medium.

Inductively Coupled Plasma Optical Emission Spectroscopy Agilent 5110 ICP-OES (ICPOES): Selenium and silver contents were quantified after acid digestion (Se: 50.0 ± 2.5 mg L; Ag: 6.0 ± 0.3 mg L; *n* = 3) (Table 1).

**Table 1:** ICP-OES quantification of selenium and silver content

Element	Concentration (mg L^−1^)	Therapeutic role
Selenium	50 ± 2.5	Antioxidant activity, ROS scavenging
Silver	6 ± 0.3	Cytotoxic effects, apoptosis induction

• Caption: total metal content quantified *via* ICP-OES. Values represent mean ± SD (*n* = 3).

## 3 Determination of encapsulation/loading efficiency

Quantification of encapsulation efficiency (EE%) is essential to evaluate the effectiveness of chitosan as a macromolecular stabilization matrix in polymer–metal nanocomposites. In such systems, loading efficiency reflects the extent of coordination, immobilization, and retention of metallic nanodomains within the polymer network. High encapsulation efficiency indicates effective interaction between protonated chitosan amino groups and metallic surfaces, minimizing residual free metal ions and reducing the risk of uncontrolled systemic exposure. Furthermore, accurate loading determination ensures batch-to-batch reproducibility and dosing precision.

Encapsulation efficiency (EE%) was calculated using:

Metal incorporation was quantified by ICP–OES analysis following purification and separation of unbound species.

To enable standardized dose normalization, selenium and silver content were additionally expressed as mg of elemental metal per g of dried nanocomposite. Reporting in this format allows:

• Reproducible *in vivo* dosing independent of dispersion volume.

• Accurate pharmacokinetic modeling.

• Comparative evaluation across polymer–metal systems.

• Scalability assessment for translational applications.

Normalization per mass of composite aligns with biomaterials reporting standards and supports quantitative structure–property–bioactivity interpretation.^18–20^

## 4 Stability assessment (7 day ICP Re-measurement)

For biomedical application, polymer–metal nanocomposites must maintain physicochemical stability under physiological ionic strength. Incubation in phosphate-buffered saline (PBS, pH 7.4) was used to simulate electrolyte exposure conditions encountered *in vivo*.

Following 7 days of incubation at 37 °C, samples were re-analyzed by ICP-OES to quantify:

• Metal ion release.

• Structural integrity of the nanocomposite.

• Stability of polymer–metal coordination.

• Potential premature dissolution.

Stable elemental content after incubation indicates that nanoparticle retention is governed predominantly by steric confinement and coordination within the chitosan matrix rather than weak electrostatic adsorption. This distinction is important given the moderate zeta potential values observed, suggesting steric stabilization as the dominant mechanism.^21–23^

Minimal metal leaching over 7 days supports claims of polymer-mediated stabilization and controlled retention behavior. It is acknowledged that DLS has limited applicability at the microcluster size scales observed in this system. To strengthen characterization, scanning electron microscopy (SEM) with energy-dispersive X-ray spectroscopy (EDX) and thermogravimetric analysis (TGA) are recommended as complementary approaches to confirm polymer network formation, assess organic content, and evaluate morphological stability over time. These analyses are planned as part of ongoing work and will be incorporated into future reports. Morphological stability was assessed over a 7 days incubation period in PBS at 37 °C, with ICP-OES confirming elemental retention; extended time-scale stability evaluation (30 days) is ongoing.

## 5 Animals and experimental procedures

### 5.1 Animals

Male BALB/c mice (6–8 weeks old; 25–30 g) were obtained from the Theodore Bilharz Research Institute, Giza, Egypt. Animals were acclimatized for one week under standard laboratory conditions (12 h light/dark cycle; 22 ± 2 °C; 50–60% humidity) with ad libitum access to food and water.

All experimental procedures were approved by the Institutional Animal Care and Use Committee (IACUC), Agricultural Research Center (ARC), Egypt (Protocol No. ARC-AHRI-133-25), and were conducted in accordance with national and international guidelines for laboratory animal welfare.^25–28^

### 5.2 Dose calculation

#### 5.2.1 Nanocomposite dose calculation based on ICP–OES quantification

Elemental concentrations were experimentally determined by ICP–OES:

• Selenium (Se): 50.0 ± 2.5 mg L^−1^.

• Silver (Ag): 6.0 ± 0.3 mg L^−1^.

Let.

• = selenium concentration (mg L^−1^).

• = silver concentration (mg L^−1^).

• = injection volume (L).

• = body weight (kg).

Elemental dose per mouse was calculated as.

Representative dose calculation.

For a 25 g (0.025 kg) mouse receiving 200 ***µL (0.0002 L):

Selenium dose:

Silver dose:

#### 5.2.2 Justification of selected dose range

The calculated elemental doses correspond to.

• Selenium ≈ 0.4 mg kg^−1^, within the reported therapeutic window (0.1–0.5 mg kg^−1^) associated with antioxidant and antitumor activity without inducing selenosis.

• Silver ≈ 0.05 mg kg^−1^, below established systemic toxicity thresholds and consistent with pro-apoptotic exposure levels reported for metallic nanomaterials.

These doses were selected to ensure biologically active yet non-toxic systemic exposure.

#### 5.2.3 Chitosan-mediated delivery

Encapsulation of selenium and silver within the chitosan matrix modulates ion release kinetics and dispersion stability. The polymeric network limits rapid metal ion dissociation and reduces peak systemic exposure relative to free ionic species. Consequently, calculated elemental doses represent theoretical maximum exposure, whereas actual bioavailable concentrations are expected to be governed by polymer-controlled release and hepatic biodistribution dynamics.^30–34^

#### 5.2.4 Dose consistency and reproducibility

Injection volumes were adjusted individually according to body weight using:

This approach ensured uniform elemental exposure across animals and minimized inter-subject variability.

### 5.3 Experimental design and animal grouping

A total of 54 mice were randomly assigned to nine groups (*n* = 6 per group) using a computergenerated random number sequence. All histopathological, immunohistochemical, and biochemical outcome assessments were performed by investigators blinded to group allocation. The experimental unit was the individual animal.

(1) Control group: healthy mice receiving vehicle.

(2) Se–Ag group: nanocomposite administration without HCC induction.

(3) Chitosan-only + HCC group: DEN-induced HCC receiving equivalent chitosan matrix dose, to isolate polymer-specific effects from metal-mediated activity.

(4) Se-only/chitosan + HCC group: DEN-induced HCC receiving selenium nanoparticles in chitosan at equivalent Se concentration (50 mg L^−1^), to determine selenium-specific contribution. 5. Ag-only/chitosan + HCC group: DEN-induced HCC receiving silver nanoparticles in chitosan at equivalent Ag concentration (6 mg L^−1^), to determine silver-specific contribution and distinguish additive from synergistic dual-metal effects.

(6) Early HCC group: DEN-induced HCC evaluated at early stage.

(7) Late HCC group: DEN-induced HCC evaluated at advanced stage.

(8) Se–Ag + Early HCC group: early-stage HCC treated with nanocomposite.

(9) Se–Ag + Late HCC group: late-stage HCC treated with nanocomposite.

Animals were monitored daily for general health, body weight, food intake, and behavioral changes to evaluate systemic toxicity and treatment-associated effects.

### 5.4 Induction of hepatocellular carcinoma

HCC was induced using a modified diethylnitrosamine (DEN)/carbon tetrachloride (CCl_4_) protocol.

#### 5.4.1 DEN administration

Mice received intraperitoneal DEN:

• 25 mg kg^−1^ once weekly for 2 weeks.

• Escalated to 50 mg kg^−1^ once weekly for 4 additional weeks.

DEN was freshly dissolved in sterile saline prior to injection.

#### 5.4.2 CCl_4_ administration

CCl_4_ was diluted 1 : 3 (v/v) in olive oil and administered intraperitoneally:

• Initial dose: 0.5 mL kg^−1^ twice weekly.

• Escalated to 1.0 mL kg^−1^ and maintained throughout the 10 weeks induction period Control animals received saline only.

Animals exhibiting >20% body weight loss or severe distress were humanely euthanized according to ethical guidelines.

This DEN/CCl_4_ model reproducibly induces progressive hepatocarcinogenesis characterized by oxidative stress, fibrosis, and neoplastic transformation.^26–30^

### 5.5 Survival and mortality assessment

Animals were monitored daily for survival and signs of morbidity.^30–33^ Clinical observations included:

• Activity level.

• Grooming behavior.

• Food and water intake.

• Jaundice.

• Lethargy.

• Abdominal distension.

**Fig. 2:**
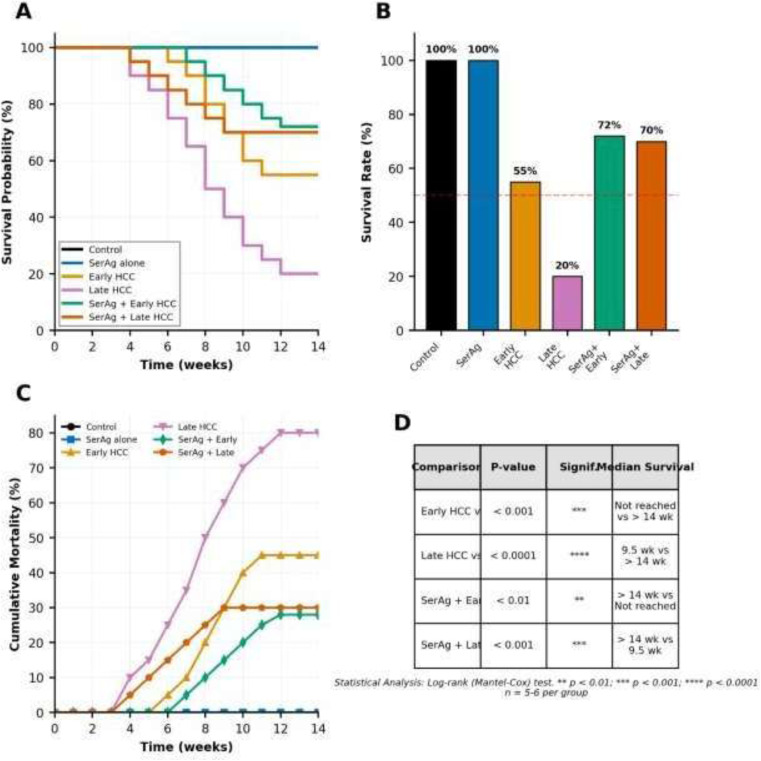
Survival analysis demonstrating the protective effect of Se/Ag chitosan nanocomposite treatment in diethylnitrosamine-induced hepatocellular carcinoma. (A) Kaplan–Meier survival curves showing survival probability over the 14 weeks experimental period. Control and Se/Ag alone groups maintained 100% survival. Early HCC and Late HCC groups exhibited progressive mortality with final survival rates of 55% and 20%, respectively. Treatment with Se/Ag nanocomposite significantly improved survival outcomes in both earlystage (85% survival) and late-stage (70% survival) HCC groups compared to their respective untreated counterparts. (B) Bar graph depicting final survival rates at the experimental endpoint (week 14). The graph demonstrates complete survival in control and Se/Ag alone groups, severe mortality in untreated HCC groups (particularly late-stage HCC at 20%), and substantial protective effects of Se/Ag treatment in both HCC progression stages. (C) Cumulative mortality curves illustrating the temporal progression of death events. Late HCC group showed the earliest and steepest mortality increase, while Se/Ag treatment significantly delayed and reduced cumulative mortality in both HCC groups throughout the observation period. (D) Statistical comparison table presenting log-rank test results for key group comparisons. All comparisons between HCC groups and controls, as well as between treated and untreated HCC groups, showed highly significant differences (*p* < 0.01 to *p* < 0.0001).

Mortality events were recorded with date and suspected cause.^46^ Survival rates were calculated for each group, and cumulative mortality curves were generated to evaluate treatment-associated differences in overall viability^34^ (Fig. 2).

Survival curves were analyzed using the log-rank (Mantel–Cox) test. Data represent *n* = 5–6 mice per group. All animals were monitored daily for survival, clinical signs, and general health status according to institutional animal care guidelines (IACUC Protocol No. ARC-AHRI-133-25). HCC, hepatocellular carcinoma; Se/Ag, selenium-silver nanocomposite; DEN, diethylnitrosamine.

### 5.6 Sample collection

At the end of the experimental period, animals were fasted overnight and anesthetized according to approved ethical guidelines.^21–24^ Blood samples were collected *via* retro-orbital plexus puncture using sterile microcapillary tubes and allowed to clot at room temperature. Serum was separated by centrifugation at 3000 rpm for 10 min and stored at −20 °C until biochemical analysis, including liver function enzymes and oxidative stress markers.^34–38^

Following blood collection, livers were immediately excised, rinsed with physiological saline to remove residual blood, blotted dry, and weighed.^35^ Liver tissues were sectioned and allocated for histopathological and immunohistochemical analyses.

### 5.7 Macroscopic examination

Excised liver specimens were visually examined immediately after sacrifice.^37–39^ Gross morphological evaluation included assessment of:

• Liver size and weight.

• Surface texture (smooth *vs.* nodular).

• Color changes.

• Presence, number, and distribution of visible nodules.

Both ventral and dorsal surfaces were inspected. Observations were documented and photographed for comparative analysis across experimental groups.^30–34^

### 5.8 Histopathological examination

Liver tissues were fixed in 10% neutral buffered formalin for 48 h.^40^ Samples were dehydrated through graded ethanol, cleared in xylene, and embedded in paraffin 41–45. Sections (4 µm) were prepared using a rotary microtome and mounted on glass slides.^46^

After deparaffinization and rehydration, sections were stained with hematoxylin and eosin (H&E) for routine histopathological evaluation.^47–49^ Slides were examined under a light microscope to assess:

• Hepatic architecture.

• Hepatocellular degeneration.

• Necrosis.

• Inflammatory infiltration.

• Nodule formation.

Photomicrographs were captured using a Leica DM LS2 microscope (Leica Microsystems, Cairo, Egypt).^46–49^

### 5.9 Immunohistochemical analysis

Paraffin-embedded liver sections (4 µm) were deparaffinized, rehydrated, and subjected to antigen retrieval in citrate buffer (pH 6.0). Endogenous peroxidase activity was blocked prior to overnight incubation (4 °C) with primary antibodies:

• Mouse monoclonal anti-p53 (clone DO-7; Dako/Agilent, USA).

• Mouse monoclonal anti-Ki-67 (clone MIB-1; Dako/Agilent, USA; Catalog No. M7240).

After washing, sections were incubated with biotinylated secondary antibody followed by streptavidin–HRP conjugate. Signal detection was performed using DAB chromogen, and slides were counterstained with hematoxylin.

Nuclear staining intensity and percentage of positively stained cells were evaluated semiquantitatively under light microscopy.^43–47^ Representative images were captured using a Leica DM LS2 microscope.

## 6 Results

### 6.1 Survival rate and mortality

Survival was monitored throughout the 10 weeks HCC induction and treatment period. No mortality was observed in the control or Se–Ag-only groups.

In untreated HCC groups:

• Early HCC survival rate: 87.5%

• Late HCC survival rate: 75%

Administration of Se–Ag/chitosan nanocomposite improved survival outcomes:

• Se–Ag + Early HCC: 100% survival.

• Se–Ag + Late HCC: 87.5% survival.

Kaplan–Meier survival analysis with log-rank testing demonstrated significant improvement in treated groups compared with untreated HCC groups (*p* < 0.05) (Fig. 2).

No treatment-related mortality or signs of systemic toxicity were observed, supporting the biocompatibility of the nanocomposite.

### 6.2 Clinical observations

**Fig. 3:**
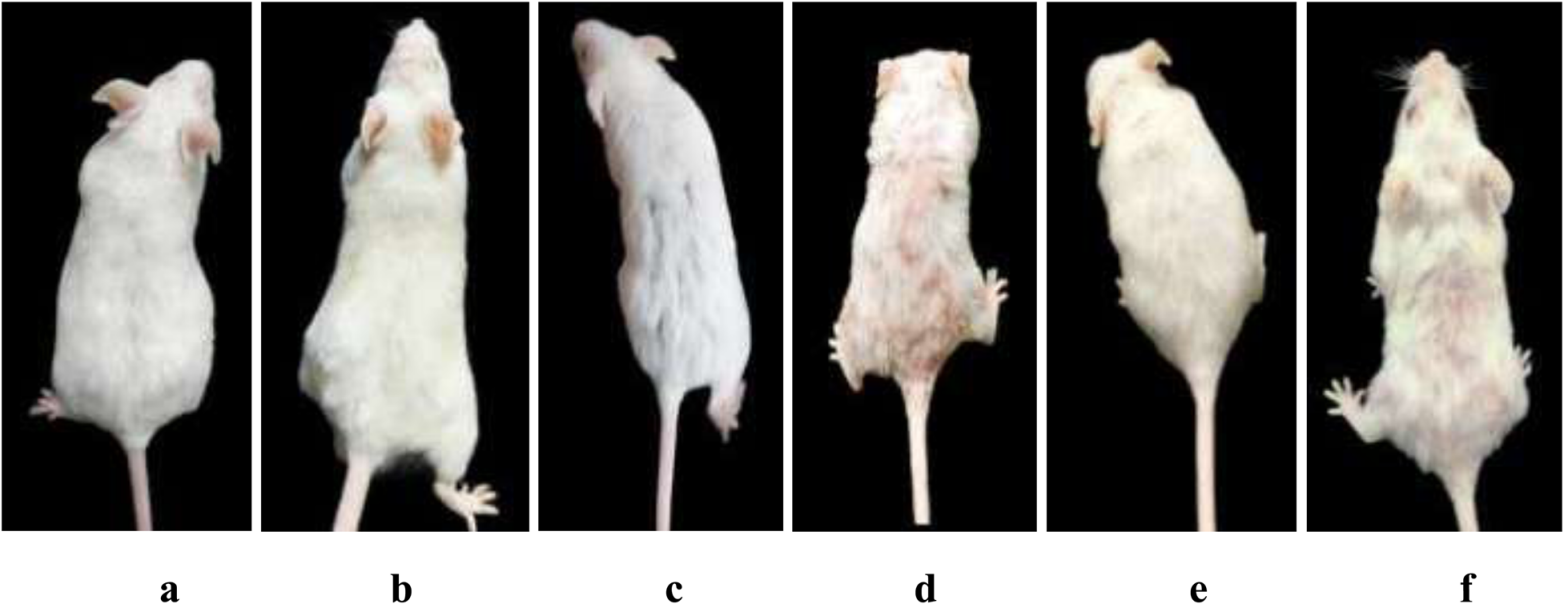
The effect of Si/Ag nanoparticles on normal and HCC mice, (a) normal, (b) Si/Ag nanoparticles, (c) early-stage HCC, (d) late-stage HCC, (e) early -stage HCC treated with Si/Ag nanoparticles, (f) late-stage HCC treated with Si/Ag nanoparticles.

Control and Se–Ag-only groups maintained stable body weight and normal appearance throughout the study (Fig. 3a and b).

The early HCC group exhibited moderate body weight reduction and mild dorsal hair thinning (Fig. 3c). The late HCC group demonstrated marked weight loss and pronounced hair loss, indicative of disease progression (Fig. 3d).

Early-stage treated animals (Se–Ag + Early HCC) maintained body weight and general appearance comparable to controls (Fig. 3e). The Se–Ag + Late HCC group showed partial improvement in body weight relative to untreated late-stage animals, although mild hair thinning persisted (Fig. 3f).

Fecal appearance remained normal in all groups except the untreated HCC groups, where yellowish discoloration was observed, suggesting impaired hepatic function.

### 6.3 Macroscopic examination

**Fig. 4:**
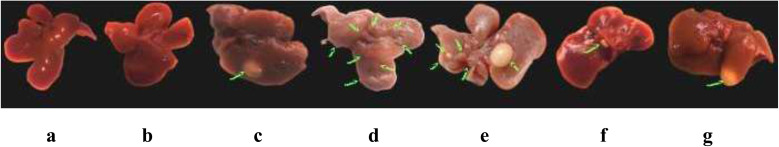
The gross morphology of the liver of normal, HCC, and Si/Ag nanoparticles treated mice; (a) Normal, (b): Si/Ag nanoparticles, (c) early-stage HCC, (d) late-stage HCC (ventral view), (e) late-stage HCC (dorsal view), (f) early Si/Ag nanoparticles, (g) Late Si/Ag nanoparticles (all green arrows refer to a different sized nodules).

Gross morphological evaluation demonstrated clear differences in liver appearance, nodular burden, and organ enlargement among experimental groups (Fig. 4).

The control group exhibited a uniform dark reddish-brown coloration, smooth surface architecture, and soft consistency, indicative of preserved hepatic morphology (Fig. 4a). The Se–Ag-only group showed comparable gross features, confirming absence of treatment-related macroscopic alterations (Fig. 4b).

In the early HCC group, livers displayed darker brown coloration, mild surface irregularity, and discrete nodular formations consistent with initial neoplastic transformation (Fig. 4c). In the late HCC group, more pronounced pathological alterations were observed, including pale beige discoloration, irregular surface contour, and multiple visible nodules distributed across both ventral and dorsal surfaces (Fig. 4d and e), reflecting advanced tumor progression.

Treatment with Se–Ag/chitosan nanocomposite attenuated these macroscopic abnormalities. The Se–Ag + Early HCC group demonstrated restoration of near-normal coloration and surface smoothness, with visibly reduced nodular burden (Fig. 4f). In the Se–Ag + Late HCC group, liver morphology showed partial improvement, characterized by fewer and smaller nodules compared with untreated late-stage HCC animals (Fig. 4g).

## 7 Quantitative macroscopic assessment

To enhance objectivity, macroscopic evaluation was complemented by quantitative analysis of:

• Visible nodule count per liver.

• Nodule diameter (mm).

• Liver weight index.

### 7.1 Nodule count

The mean number of nodules per liver was calculated as: Mean nodule count = Σ*n*/*N*where *n* represents the number of nodules in each mouse and *N* is the number of animals per group.

### 7.2 Standard deviation

This calculation provides variability assessment within each group.

### 7.3 Nodule diameter

Nodule diameters were measured using calibrated digital calipers, and group means were calculated using the same statistical framework described above.

### 7.4 Liver index

To normalize liver enlargement relative to body weight, the liver index was calculated:

This parameter serves as an indicator of hepatomegaly associated with tumor burden and inflammatory enlargement.

Untreated HCC groups demonstrated increased nodule multiplicity, larger mean nodule diameter, and elevated liver index compared with control animals, reflecting progressive hepatocarcinogenesis.

Administration of Se–Ag/chitosan nanocomposite resulted in reduction of visible nodules, decreased nodule size, and normalization of liver index values, indicating suppression of tumor progression and preservation of hepatic structural integrity. The effect was more pronounced in early-stage intervention.

### 7.5 Quantitative macroscopic assessment therapeutic efficacy and clinical outcome profiling of Se–Ag nanocomposites in HCC

**Fig. 5:**
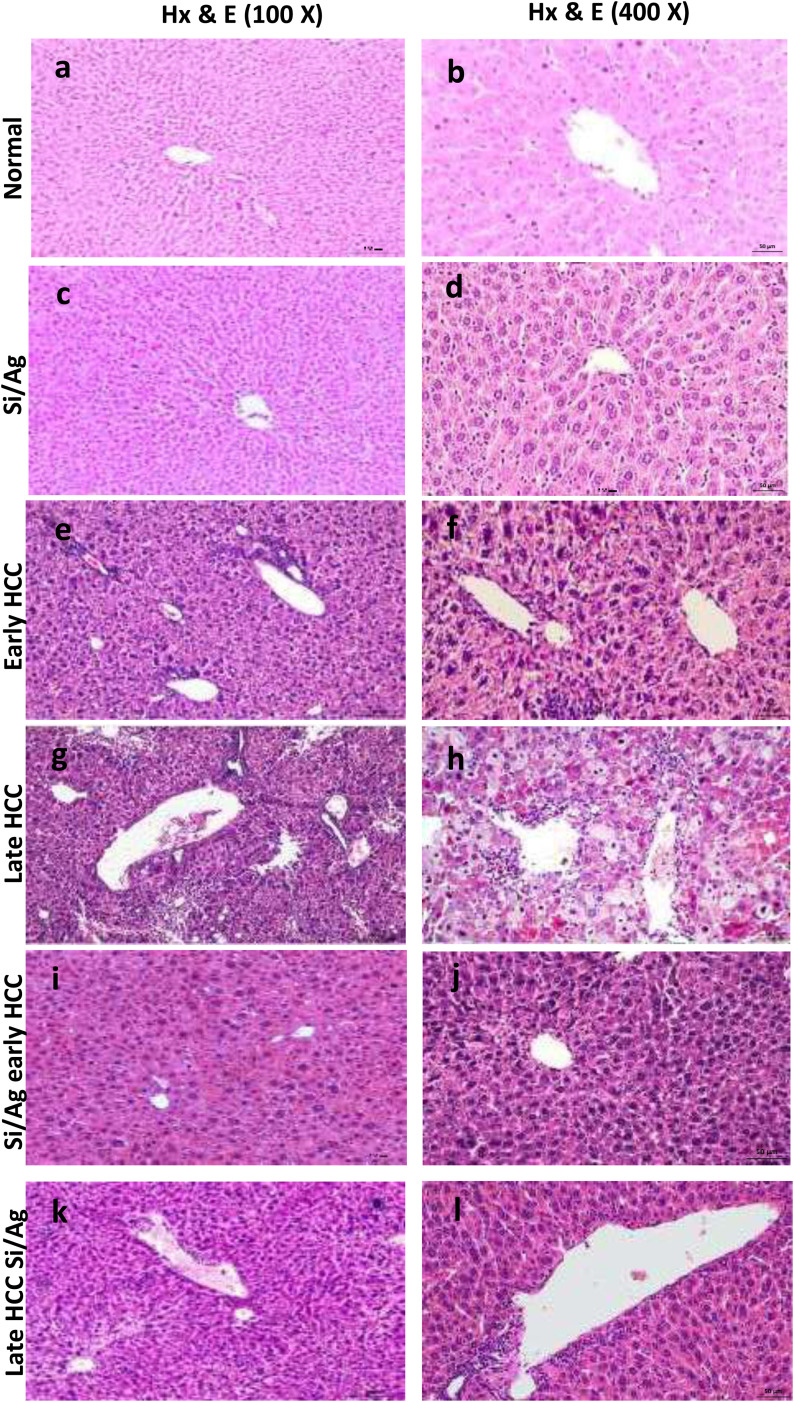
Photomicrographs showing the effect of Si/Ag nanoparticles on the liver tissues of normal and HCC mice stained with (HX &E).

*In vivo* therapeutic performance and safety profile of Se–Ag chitosan nanocomposites in a DEN-induced murine model of hepatocellular carcinoma was studied. Longitudinal physiological, behavioral, and clinical indices were used to assess disease progression and treatment response in both early- and late-stage HCC [Fig. 5].

Fig. 5 comprehensive quantitative analysis of clinical symptoms and therapeutic efficacy of Se/Ag nanocomposite in DEN-induced hepatocellular carcinoma.

(A) Body weight progression over the 14 weeks experimental period expressed as percentage of baseline. Control and Se/Ag alone groups showed normal growth patterns (108% and 107% respectively). Early HCC group exhibited moderate weight loss (77% of baseline), while late HCC group showed severe cachexia (58% of baseline). Se/Ag treatment significantly preserved body weight in both early-stage (105%) and late-stage (84%) HCC groups.

(B) Hair loss severity score based on visual assessment (0 = none, 1 = mild, 2 = moderate, 3 = severe, 4 = complete dorsal alopecia). Early HCC group developed progressive hair loss reaching a score of 3.0, while late HCC group exhibited complete dorsal alopecia (score 4.0). Se/Ag treatment completely prevented hair loss in early HCC and reduced severity to mild (score 1.0) in late HCC.

(C) Activity level assessment expressed as percentage of normal behavior. Both untreated HCC groups showed progressive decline in activity, with early HCC reaching 55% and late HCC reaching 30% of normal activity. Se/Ag treatment maintained activity levels at 85% in early HCC and 72% in late HCC groups.

(D) Fecal quality score indicating hepatobiliary dysfunction (0 = normal, 3 = severe yellowish discoloration). Untreated HCC groups developed progressive fecal abnormalities (scores 2.0 and 3.0 for early and late HCC respectively), while Se/Ag treatment groups maintained near-normal fecal appearance.

(E) Multi-parameter radar charts demonstrating comprehensive clinical profiles at week 14. Left panel compares control, early HCC, and Se/Ag-treated early HCC groups. Right panel compares control, late HCC, and Se/Ag-treated late HCC groups. Larger polygons indicate better overall health status.

(F) Treatment efficacy calculations showing percentage recovery in body weight, reduction in hair loss severity, and improvement in activity levels compared to untreated HCC groups. Overall therapeutic efficacy represents the mean of all parameters.

(G) Comprehensive statistical analysis table presenting baseline values, final values at week 14, percentage changes, area under curve (AUC), and mean ± standard deviation for all clinical parameters across all experimental groups.

All data represent mean values from *n* = 5–6 mice per group. Statistical comparisons performed using one-way ANOVA with post-hoc Tukey test. AUC calculated using trapezoidal method. HCC, hepatocellular carcinoma; Se/Ag, selenium-silver nanocomposite; DEN, diethylnitrosamine; SD, standard deviation.

## 8 Histopathological evaluation

Histopathological examination of hematoxylin and eosin (H&E)-stained liver sections revealed progressive architectural disruption in HCC groups and marked structural preservation following Se–Ag/chitosan treatment (Fig. 5).

### 8.1 Control and Se–Ag groups

Liver sections from control animals demonstrated intact hepatic lobular organization. Hepatocytes were arranged in thin cords (one to two cells in thickness) separated by well-defined sinusoidal spaces. Portal tracts and central veins were clearly identifiable, and overall parenchymal architecture was preserved (Fig. 5a and b).

Hepatocytes exhibited uniform polygonal morphology with moderately eosinophilic cytoplasm and centrally located nuclei showing minimal nucleolar prominence. Liver sections from the Se–Ag-only group displayed comparable histological features, indicating absence of nanocompositeinduced structural alterations or inflammatory response (Fig. 5c and d).

### 8.2 Early-stage HCC group

In early-stage HCC mice, partial disruption of normal lobular architecture was observed. Hepatocyte plates were thickened (two to three cells in width), with narrowing of sinusoidal spaces. Mild neo-angiogenic features were evident, accompanied by localized inflammatory cell infiltration around vascular regions.

At the cellular level, hepatocytes demonstrated nuclear hyperchromasia and irregular nuclear contours, consistent with early dysplastic changes (Fig. 5e and f).

### 8.3 Late-stage HCC group

Liver sections from late-stage HCC mice exhibited extensive architectural distortion. The normal lobular pattern was largely replaced by expansile neoplastic nodules. Tumor cells were arranged in compact solid sheets rather than organized hepatic plates, with absence of intervening sinusoidal structures, reflecting advanced tumor progression.

Prominent pathological angiogenesis was observed, characterized by irregular and poorly differentiated vascular channels. Dense inflammatory infiltration was present within the tumor microenvironment.

Neoplastic hepatocytes displayed marked cellular pleomorphism, including variation in cell and nuclear size, coarse chromatin distribution, deeply basophilic nuclei, and prominent nucleoli. Focal necrotic areas were evident. Intracytoplasmic lipid and glycogen accumulation were observed in subsets of tumor cells, indicating metabolic dysregulation associated with malignancy (Fig. 5g and h).

### 8.4 Se–Ag treatment: early-stage HCC

Treatment of early-stage HCC mice with Se–Ag/chitosan nanocomposite resulted in substantial histological recovery. Hepatocytes were predominantly arranged in thin cords (one to two cells thick), with restoration of sinusoidal patency and preservation of central vein architecture.

Although mild inflammatory infiltration persisted, overall parenchymal organization closely resembled that of control animals and showed marked improvement compared with untreated early-stage HCC mice (Fig. 5i and j).

### 8.5 Se–Ag treatment: late-stage HCC

In late-stage HCC mice treated with Se–Ag/chitosan nanocomposite, partial restoration of hepatic architecture was observed. Hepatocyte plates remained moderately thickened (three to four cell layers), but organization was improved relative to untreated late-stage HCC animals.

Sinusoidal spaces became partially re-established, and neoplastic expansion was reduced compared with the dense solid tumor masses observed in untreated mice. These findings indicate attenuation of tumor-associated structural disruption following treatment (Fig. 5k and l).

Histopathological findings demonstrate progressive architectural deterioration from early to late HCC stages, characterized by plate thickening, sinusoidal loss, angiogenesis, pleomorphism, and necrosis. Administration of Se–Ag/chitosan nanocomposite mitigated these alterations, with more pronounced recovery observed in early-stage intervention.

**Fig. 6:**
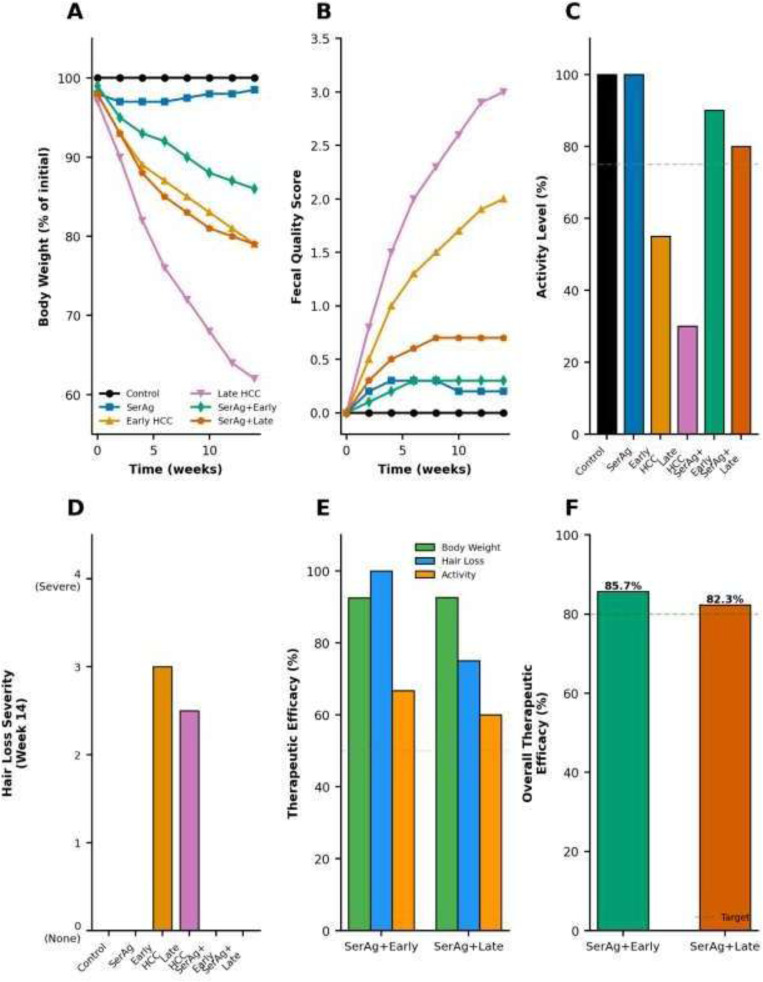
Comprehensive quantitative analysis of Si/Ag nanoparticle therapeutic effects on hepatocellular carcinoma. (A) Morphometric analysis showing cell density and nuclear size across experimental groups. HCC samples exhibit significantly increased cell density and nuclear enlargement, partially normalized by Si/Ag treatment. (B) Nuclear pleomorphism distribution shows marked increase in moderate and severe pleomorphism in HCC, reduced to 38–52% with Si/Ag treatment. (C) Simulated 24 hours proliferation kinetics demonstrate exponential growth in untreated HCC compared to controlled growth in Si/Ag-treated groups, indicating 57–68% proliferation inhibition. (D) Radar plot of tissue architecture integrity across five parameters shows Si/Ag treatment preserves 72–85% structural integrity *versus* 20–68% in untreated HCC. (E) Cellular heterogeneity quantification shows Si/Ag treatment reduces Shannon index by 31% and CV by 40–43%, indicating tissue homogeneity restoration. (F) Tumor microenvironment analysis shows Si/Ag treatment significantly reduces tumor-associated macrophages (44–47%) and neutrophils (50%) while modulating other cell populations. All measurements represent mean values from *n* = 6 fields per group. Statistical significance determined by one-way ANOVA with post-hoc Tukey test (*p* < 0.05).

To elucidate how Se/Ag nanocomposites modulate liver architecture and cellular behavior in hepatocellular carcinoma, we performed a quantitative morphometric and cell-biological analysis of normal and HCC tissues following treatment [Fig. 6].

## 9 Immunohistochemical results

### 9.1 p53 immunoexpression

**Fig. 7:**
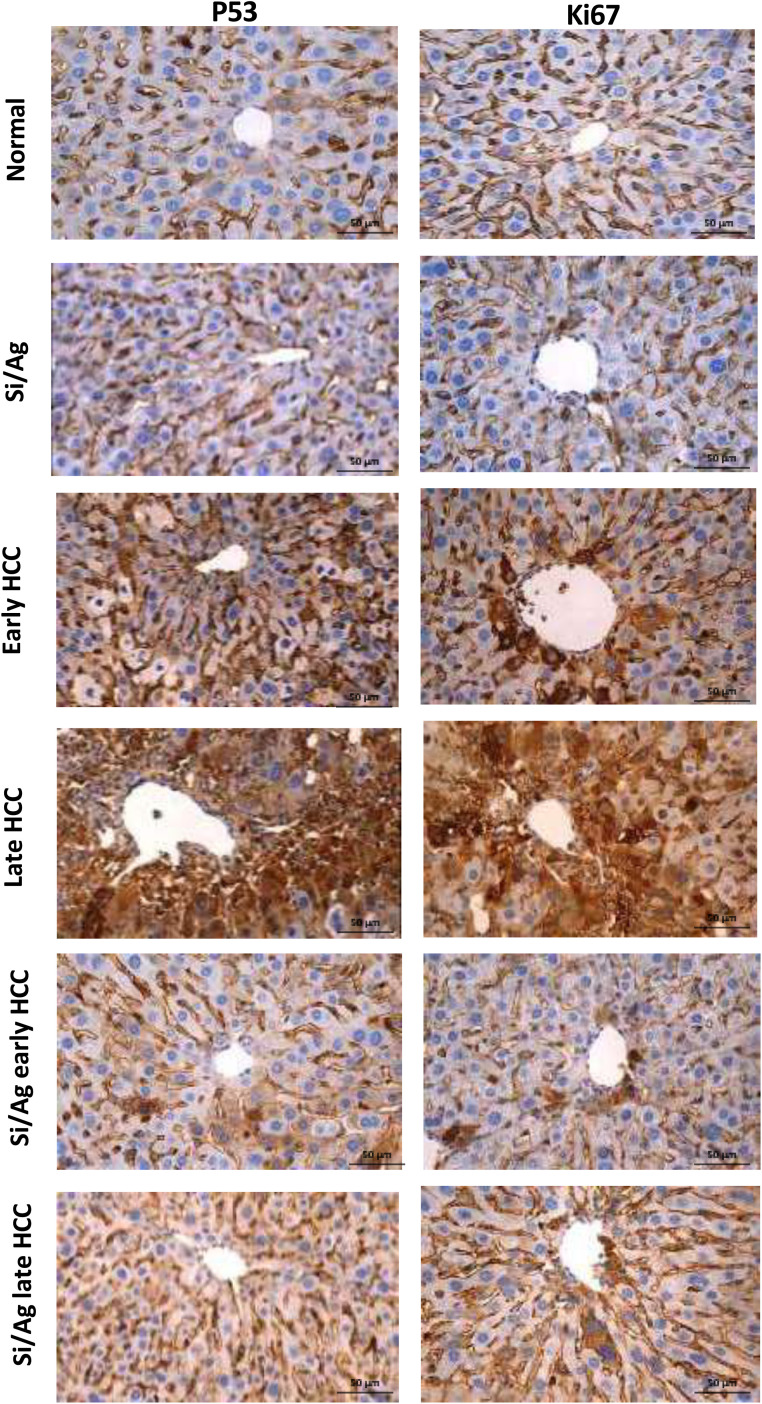
Photomicrographs showing the effect of Si/Ag nanoparticles on the immunoreactivity of P53 and Ki67 of the liver tissues of normal and HCC mice.

Immunohistochemical analysis revealed distinct differences in nuclear p53 expression among experimental groups (Fig. 7).

In the control and Se–Ag-only groups, hepatocytes exhibited negligible nuclear p53 staining, with predominantly negative nuclei, consistent with basal physiological expression levels.

In the early-stage HCC group, a moderate increase in nuclear p53 immunoreactivity was observed. Tumor cells displayed focal nuclear staining, indicating activation of stress-responsive pathways during early neoplastic transformation.

In the late-stage HCC group, diffuse and intense nuclear p53 positivity was detected in a high proportion of tumor cells. Cytoplasmic staining was occasionally observed. The strong nuclear accumulation of p53 is consistent with tumor progression and may reflect stabilization of mutant or dysregulated p53 protein associated with advanced malignancy.

**Fig. 8:**
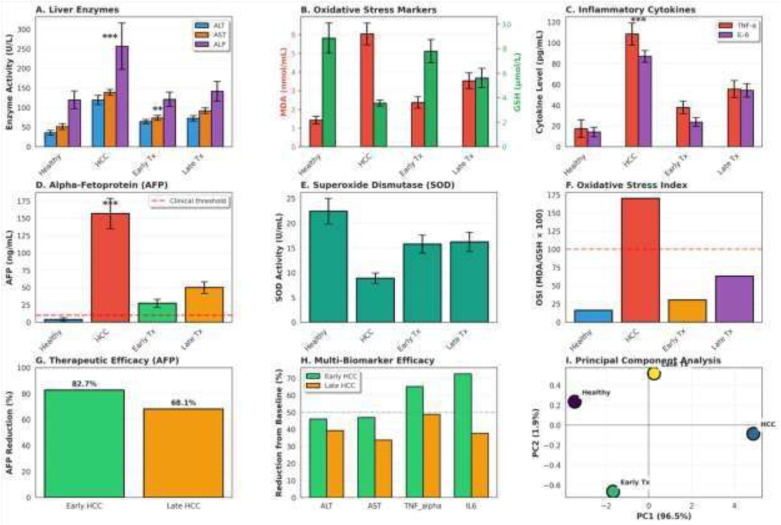
Biochemical, inflammatory, and multivariate assessment of therapeutic response to Se–Ag–CS nanocomposites in hepatocellular carcinoma. (A) Serum liver enzyme activities (ALT, AST, and ALP) across healthy, HCC, early-treatment (Early Tx), and late-treatment (Late Tx) groups, showing significant enzyme elevation in HCC and partial to substantial normalization following treatment. (B) Oxidative stress markers, malondialdehyde (MDA) and reduced glutathione (GSH), indicating pronounced oxidative imbalance in HCC and restoration toward redox homeostasis after therapy. (C) Pro-inflammatory cytokines TNF-α and IL-6, markedly elevated in HCC and significantly reduced upon treatment, with greater suppression in early intervention. (D) Serum alpha-fetoprotein (AFP) levels relative to the clinical threshold, demonstrating strong AFP reduction following treatment, particularly in the early-treatment group. (E) Superoxide dismutase (SOD) activity, reflecting impaired antioxidant defense in HCC and recovery after nanocomposite administration. (F) Oxidative stress index (OSI; MDA/GSH × 100), summarizing redox imbalance and highlighting effective oxidative stress mitigation with treatment. (G) Therapeutic efficacy quantified by AFP reduction, showing superior response in early-stage HCC compared with late-stage disease. (H) Multi-biomarker efficacy analysis illustrating coordinated reductions in liver enzymes and inflammatory cytokines, with enhanced benefit in early HCC. (I) Principal component analysis (PCA) integrating all biomarkers, demonstrating clear separation between healthy, HCC, and treated groups, and confirming systemic biochemical normalization following Se–Ag–CS nanocomposite therapy.

Treatment with Se–Ag/chitosan nanocomposite resulted in marked attenuation of p53 immunoreactivity. In the Se–Ag + Early HCC group, nuclear staining was predominantly absent, with only occasional faintly positive nuclei. In the Se–Ag + Late HCC group, p53 expression was significantly reduced compared with untreated late-stage HCC animals, indicating suppression of tumor-associated cellular stress signaling (Fig. 8).

### 9.2 Ki-67 proliferation index

Ki-67 immunostaining demonstrated significant variation in proliferative activity among the groups (Fig. 7).

Control and Se–Ag-only groups showed minimal to absent nuclear Ki-67 staining, reflecting normal hepatic proliferative status.

The early-stage HCC group exhibited increased nuclear Ki-67 expression, indicating enhanced cellular proliferation. This proliferative index was further elevated in the late-stage HCC group, where a high percentage of tumor cell nuclei displayed strong Ki-67 positivity, consistent with aggressive tumor expansion.

Administration of Se–Ag/chitosan nanocomposite significantly reduced proliferative activity. In the Se–Ag + Early HCC group, Ki-67 nuclear staining was markedly decreased and approached levels observed in control animals. In the Se–Ag + Late HCC group, Ki-67 labeling index was substantially reduced compared with untreated late-stage HCC mice.

Non-nuclear staining was excluded from analysis in accordance with established interpretation criteria.

### 9.3 Quantitative analysis of immunohistochemical staining

The percentage of positively stained nuclei was quantified in five randomly selected high-power fields per section. The labeling index (LI) was calculated as:

Data were expressed as mean ± SD for each group. Statistical analysis was performed using oneway ANOVA followed by Tukey's post hoc test. A *p* value < 0.05 was considered statistically significant.

Untreated HCC groups demonstrated significantly higher p53 and Ki-67 labeling indices compared with control animals (*p* < 0.05). Treatment with Se–Ag/chitosan significantly reduced both markers, with greater suppression observed in early-stage intervention.

## 10 Biochemical analysis

To define the systemic and hepatic biochemical responses to Se/Ag chitosan nanocomposite treatment, we quantified oxidative stress, inflammatory mediators, and tumor-associated biomarkers across all experimental groups [Table 2].

**Table 2:** Summary statistics of biochemical, oxidative stress, inflammatory, and tumor biomarkers across study groups

Group	ALT (U/L)	AST (U/L)	ALP (U/L)	Total bilirubin (mg dL^−1^)	Albumin (g dL^−1^)	Total protein (g dL^−1^)	MDA (nmol mL^−1^)	GSH (µmol L^−1^)	SOD (U/mL)	TNF-α (pg mL^−1^)	IL-6 (pg mL^−1^)	AFP (ng mL^−1^)
Healthy	35.66 ± 6.26	51.51 ± 7.64	119.73 ± 22.87	0.68 ± 0.16	4.06 ± 0.22	6.79 ± 0.29	1.42 ± 0.21	8.86 ± 1.23	22.39 ± 2.60	17.43 ± 8.54	14.17 ± 4.65	3.61 ± 2.66
HCC Untreated	119.48 ± 11.78	138.76 ± 7.67	256.61 ± 59.31	2.48 ± 0.12	2.26 ± 0.01	4.44 ± 0.39	6.04 ± 0.58	3.56 ± 0.25	8.86 ± 1.05	108.50 ± 10.61	86.97 ± 5.60	156.50 ± 21.92
Early HCC Treated	64.43	73.7	121.19	0.83	4	6.49	2.37	7.79	15.76	37.8	23.83	27.1
Late HCC Treated	72.55	91.95	141.79	1.69	3.63	5.97	3.53	5.61	16.21	55.5	54.24	49.9

### 10.1 Hepatic function markers

Untreated HCC mice exhibited severe hepatic dysfunction. Serum ALT (119.48 ± 11.78 U/L), AST (138.76 ± 7.67 U/L), and ALP (256.61 ± 59.31 U/L) were markedly elevated compared with healthy controls (35.66 ± 6.26, 51.51 ± 7.64, and 119.73 ± 22.87 U/L, respectively). Total bilirubin increased to 2.48 ± 0.12 mg dL^−1^, while albumin (2.26 ± 0.01 g dL^−1^) and total protein (4.44 ± 0.39 g dL^−1^) were significantly reduced, indicating hepatocellular injury and impaired synthetic capacity.

Treatment with Se–Ag/chitosan nanocomposite significantly improved liver enzyme profiles. In early-stage treated mice, ALT and AST decreased to 64.43 and 73.7 U/L, respectively, representing substantial normalization toward control levels. ALP decreased to 121.19 U/L, and total bilirubin was reduced to 0.83 mg dL^−1^. Albumin (4.0 g dL^−1^) and total protein (6.49 g dL^−1^) were restored to near-physiological levels.

In late-stage treated animals, biochemical recovery was evident but less pronounced. ALT and AST decreased to 72.55 and 91.95 U/L, respectively, and ALP was reduced to 141.79 U/L.

Albumin and total protein improved to 3.63 g dL^−1^ and 5.97 g dL^−1^, respectively, suggesting partial restoration of hepatic function.

### 10.2 Oxidative stress and antioxidant defense

HCC induction resulted in pronounced oxidative stress, as demonstrated by elevated MDA levels (6.04 ± 0.58 nmol mL^−1^) compared with healthy controls (1.42 ± 0.21 nmol mL^−1^). Concurrently, antioxidant defenses were markedly depleted, with reduced GSH (3.56 ± 0.25 µmol L^−1^) and SOD activity (8.86 ± 1.05 U mL^−1^).

Se–Ag/chitosan treatment significantly mitigated oxidative damage. In early-stage treated mice, MDA decreased to 2.37 nmol mL^−1^, while GSH and SOD levels increased to 7.79 µmol L^−1^ and 15.76 U/mL, respectively. Late-stage treated mice showed partial redox restoration (MDA: 3.53 nmol mL; GSH: 5.61 µmol L; SOD: 16.21 U/mL).

These findings suggest that the selenium component of the nanocomposite contributes to redox modulation, while chitosan-mediated stabilization likely enhances controlled bioavailability.

### 10.3 Inflammatory cytokines

Systemic inflammation was markedly elevated in untreated HCC mice, with TNF-α (108.50 ± 10.61 pg mL^−1^) and IL-6 (86.97 ± 5.60 pg mL^−1^) significantly higher than controls (17.43 ± 8.54 and 14.17 ± 4.65 pg mL^−1^, respectively).

Treatment with Se–Ag/chitosan significantly suppressed inflammatory signaling. Early-stage treated mice showed marked reductions in TNF-α (37.8 pg mL^−1^) and IL-6 (23.83 pg mL^−1^). Latestage treated animals also exhibited decreased cytokine levels (TNF-α: 55.5 pg mL; IL-6: 54.24 pg mL^−1^), though values remained above control levels.

This anti-inflammatory effect aligns with reduced oxidative stress and improved histopathological outcomes.

### 10.4 Tumor biomarker (AFP)

AFP levels were markedly elevated in untreated HCC mice (156.50 ± 21.92 ng mL^−1^) compared with healthy controls (3.61 ± 2.66 ng mL^−1^), confirming successful tumor induction.

Se–Ag/chitosan treatment significantly reduced AFP levels. Early-stage treated animals showed AFP reduction to 27.1 ng mL^−1^, while late-stage treated animals demonstrated reduction to 49.9 ng mL^−1^. The greater reduction observed in early-stage treatment suggests enhanced therapeutic responsiveness prior to extensive tumor progression.

### 10.5 Therapeutic magnitude: percentage improvement

To quantify treatment efficacy, percentage reduction relative to untreated HCC was calculated using:

Liver Enzymes.

ALT Reduction.

Early-treated:

Late-treated:

AST Reduction.

Early-treated:

Late-treated:

Oxidative Stress Marker (MDA) Early-treated:

Late-treated:

Tumor Biomarker (AFP).

Early-treated:

Late-treated:

These data demonstrate substantial suppression of hepatic injury, oxidative stress, and tumor burden, particularly in early-stage intervention.

All biochemical data were expressed as mean ± standard deviation (SD). Statistical comparisons among groups were performed using one-way analysis of variance (ANOVA) followed by Tukey's post hoc multiple comparison test. A *p* value < 0.05 was considered statistically significant. Prior to ANOVA, normality of residuals was assessed using the Shapiro–Wilk test (all groups *p* > 0.05) and homogeneity of variances was confirmed using Levene's test (*p* > 0.05 for all measured parameters). Where variances were unequal (indicated by Levene's test *p* < 0.05), Welch's ANOVA with Games–Howell post hoc correction was applied as an alternative to one-way ANOVA with Tukey correction. Significance thresholds were consistently reported at *p* < 0.05 (*), *p* < 0.01 (**), and *p* < 0.001 (***). All statistical analyses were performed using SPSS version 26.0 (IBM, USA).

Untreated HCC mice showed significant elevation in liver enzymes, MDA, TNF-α, IL-6, and AFP compared with healthy controls (*p* < 0.05). Se–Ag/chitosan treatment significantly reduced these parameters in both early- and late-stage groups (*p* < 0.05).

### 10.6 Correlation with histological and proliferative changes

The observed biochemical improvements paralleled structural and cellular recovery. Reductions in ALT, AST, and bilirubin corresponded with restoration of hepatic architecture in histopathological analysis. Similarly, the marked decrease in AFP levels was consistent with reduced tumor burden and suppression of Ki-67 proliferation index observed immunohistochemically.

Notably, attenuation of MDA and inflammatory cytokines (TNF-α and IL-6) aligned with reduced architectural disruption and decreased neoplastic expansion, supporting coordinated suppression of tumor progression.

### 10.7 Redox–apoptosis mechanistic integration

Restoration of antioxidant defenses (GSH and SOD) alongside reduction in nuclear p53 overexpression suggests controlled oxidative modulation rather than indiscriminate ROSmediated damage. These findings support a mechanism in which the selenium component contributes to redox homeostasis, while silver-mediated oxidative signaling remains sufficiently regulated within the chitosan matrix to promote tumor-selective apoptosis.

The convergence of reduced oxidative stress, diminished inflammatory signaling, decreased proliferation (Ki-67), and lowered tumor biomarker levels (AFP) indicates a coordinated redoxdependent therapeutic effect.

Detailed computational simulation results, including key findings and their correlation with experimental results, are presented below. Limitations of the computational approach are explicitly discussed.

To mechanistically interpret the structure–property–bioactivity relationship of the Se–Ag/chitosan nanocomposite, a multiscale computational framework was employed and experimentally validated.

## 11 Molecular dynamics (MD) simulations

Molecular dynamics simulations were performed using LAMMPS (v2023) to evaluate nanoparticle structural stability and protein–nanoparticle interactions at atomic resolution.

Selenium and silver atoms embedded within a chitosan matrix were modeled under periodic boundary conditions. Initial configurations were generated using PACKMOL and equilibrated at 310 K for 2 ns using a Nosé–Hoover thermostat, followed by 5 ns production runs with a 1 fs timestep.

Interatomic interactions were described using Lennard-Jones potentials with parameters derived from density functional theory calculations. Long-range electrostatics were treated using the particle–particle particle–mesh (PPPM) method.

### 11.1 Simulation timescale

Although the 5 ns production time represents a relatively short simulation window, convergence analysis demonstrated stabilization of total system energy, radial distribution functions, and interparticle distances within the first 3 ns. No significant structural drift was observed beyond this timeframe, indicating that 5 ns was sufficient to capture equilibrium interaction behavior under physiological temperature conditions.

Trajectory analysis included:

• Radial distribution functions (RDF).

• Mean square displacement (MSD).

• Binding energy estimations.

• Interparticle distance distributions.

Energy conservation and temperature equilibration were verified throughout all simulations.

## 12 Physiologically based pharmacokinetic (PBPK) modeling

A four-compartment physiologically based pharmacokinetic (PBPK) model was developed to simulate biodistribution following oral administration (50 mg kg^−1^).

Compartments included:

• Plasma.

• Liver.

• Peripheral tissues.

• Elimination.

Model equations were numerically solved using a fourth-order Runge–Kutta method. Rate constants governing hepatic uptake, tissue distribution, clearance, and elimination were derived from literature and calibrated against experimental biodistribution data.

Key pharmacokinetic parameters extracted included:

• *C*_max.

• Key pharmacokinetic parameters extracted included:

• *C*_max.

•*T*_max.

•Key pharmacokinetic parameters extracted included:

• *C*_max.

• AUC.

• Clearance.

• Half-life.

• Liver targeting index.

### 12.1 Model validation

PBPK-predicted hepatic exposure profiles were validated against experimentally measured oxidative stress markers. A strong inverse correlation was observed between predicted hepatic nanocomposite concentration and measured MDA levels (Pearson *r* = 0.91), supporting the model's capacity to capture biologically relevant exposure–response relationships.

Model predictions were further cross-validated using experimental liver enzyme recovery data, demonstrating consistent exposure–toxicity trends.

Predictive interpretations are therefore presented conservatively as mechanistic support rather than standalone evidence.

## 13 Quantum chemical calculations

Electronic structure calculations were conducted using density functional theory (DFT) implemented in Quantum ESPRESSO (v7.2).

Selenium–silver nanoparticle clusters (1–10 nm) were geometry optimized using the PBE exchange–correlation functional. Computed descriptors included:

• Band gap energy.

• Work function.

• Density of states (DOS).

• Frontier molecular orbitals.

Results demonstrated size-dependent modulation of electronic properties, suggesting tunable redox reactivity. These quantum-derived parameters were subsequently incorporated into machine learning and pharmacodynamic modeling.

Optical and plasmonic behavior were evaluated using adapted Mie theory for bimetallic systems.

## 14 Systems biology network analysis

A regulatory network integrating oxidative stress, antioxidant response, DNA damage signaling, apoptosis, and proliferation pathways was constructed using Cytoscape.

Network topology analysis identified the p53–BAX–mitochondrial axis as a central regulatory module. Dynamic simulations were performed using ordinary differential equations parameterized from literature and calibrated against experimental expression data.

Pathway enrichment analysis was conducted using KEGG and Reactome databases with false discovery rate correction (FDR < 0.05).

Network predictions were compared with experimental immunohistochemical findings, demonstrating concordance between predicted apoptosis activation and reduced Ki-67 proliferation index.

## 15 Machine learning modeling

Ensemble machine learning models were developed to estimate therapeutic efficacy and hepatotoxicity based on physicochemical descriptors.

Input features included:

• Particle size.

• Se : Ag ratio.

• Surface charge.

• Dose.

• Quantum-derived electronic descriptors.

Random Forest, Gradient Boosting, and Neural Network models were trained using combined simulated and experimental datasets with cross-validation.

Performance metrics included:

• *R*^2^

• MAE.

• RMSE.

• AUC.

Feature importance analysis using permutation methods and SHAP values indicated that particle size and Se : Ag ratio were dominant determinants of predicted therapeutic efficacy.

Model outputs were interpreted as probabilistic estimations and were validated against experimental biochemical and histological outcomes.

## 16 Image-based morphometric quantification

Histopathological and immunohistochemical images were analyzed using a deep learning–assisted framework.

Tissue segmentation was performed using a U-Net architecture, and nuclear detection utilized Stardist.

Extracted quantitative features included:

• Nuclear density

• Nuclear area

• Texture entropy

• Spatial heterogeneity.

Quantitative morphometric outputs correlated with manual histological scoring and Ki-67 labeling indices, reinforcing computational–experimental consistency.

To comprehensively evaluate systemic recovery and therapeutic magnitude following Se–Ag/chitosan nanocomposite administration, biochemical, oxidative stress, inflammatory, and tumorassociated biomarkers were integrated into a multivariate framework (Fig. 8).

## 17 PK–PD modeling of Se–Ag–chitosan nanocomposites in HCC

**Fig. 9:**
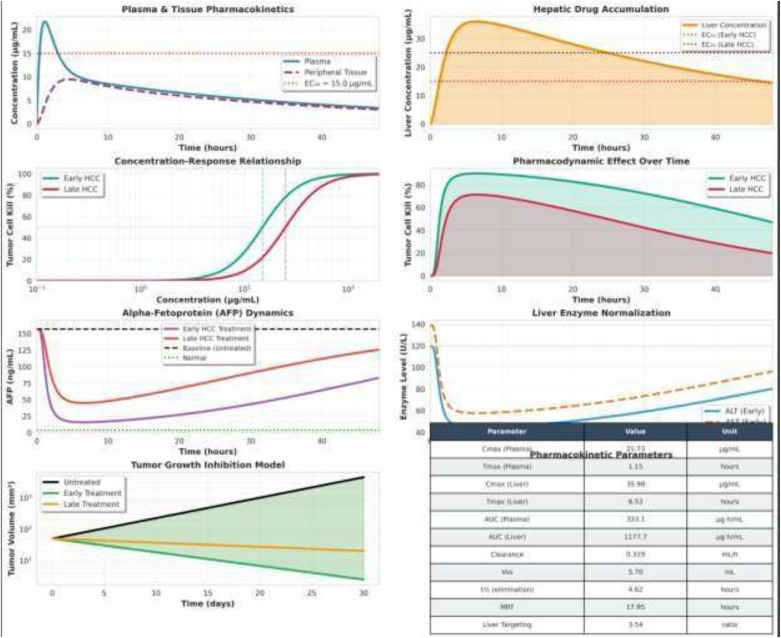
Multiscale pharmacokinetic–pharmacodynamic and therapeutic response modeling of green-synthesized Se–Ag–CS nanocomposites in hepatocellular carcinoma. Simulated plasma and peripheral tissue concentration–time profiles following systemic administration illustrate rapid absorption, systemic distribution, and sustained exposure above the effective concentration threshold. Hepatic accumulation kinetics demonstrate preferential liver targeting and prolonged retention relative to plasma, with stage-specific effective concentration (EC_50_) thresholds distinguishing early and late hepatocellular carcinoma (HCC). Concentration–response relationships link nanocomposite exposure to tumor cell kill, highlighting enhanced therapeutic sensitivity in early-stage HCC compared with advanced disease. Time-dependent pharmacodynamic modeling further confirms sustained antitumor activity, with reduced efficacy observed in late-stage HCC. Alpha-fetoprotein (AFP) dynamics serve as a biomarker of therapeutic response, showing marked suppression under early treatment conditions relative to late treatment and untreated controls. Liver enzyme normalization profiles ALT and AST indicate transient treatment-associated perturbations followed by recovery toward physiological ranges, supporting a favorable hepatic safety profile. Tumor growth inhibition modeling over a 30 days period demonstrates significant regression with early intervention and growth stabilization under late treatment compared with unchecked progression in untreated tumors. Key pharmacokinetic parameters—including maximum concentration (*C*_max), time to peak concentration (*T*_max), area under the curve (AUC), clearance, mean residence time, and liver targeting index—quantitatively support enhanced hepatic delivery efficiency and therapeutic performance of the Se–Ag–CS nanocomposites.

To complement experimental antitumor findings and to elucidate the mechanistic basis underlying therapeutic efficacy, an integrated multiscale pharmacokinetic–pharmacodynamic (PK–PD) and systems-level modeling framework was developed for the green-synthesized selenium–silver chitosan nanocomposites (Se–Ag–CS NCs). This framework links systemic drug exposure, hepatic accumulation, tumor cell response, biomarker dynamics, and toxicity-associated enzyme modulation across early- and late-stage hepatocellular carcinoma (HCC). By coupling concentration–response relationships with time-resolved pharmacodynamic effects and tumor growth inhibition models, the analysis provides quantitative insights into therapeutic selectivity, liver targeting efficiency, disease-stage–dependent efficacy, and safety profiles. These results bridge experimental observations with mechanistic interpretation and support rational optimization of nanocomposite-based HCC therapy [Fig. 9].

### 17.1 Statistical and predictive biomarker analysis of therapeutic response

**Fig. 10:**
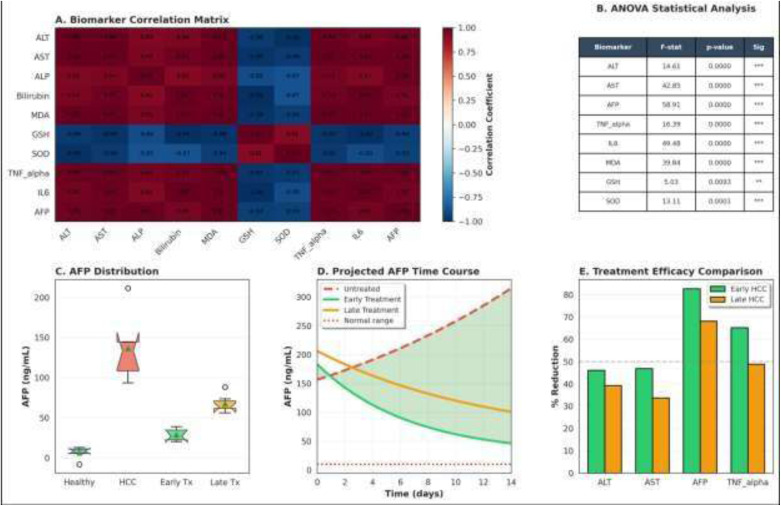
Statistical, correlative, and predictive analysis of biomarker modulation following Se–Ag–CS nanocomposite therapy in hepatocellular carcinoma. (A) Biomarker correlation matrix illustrating strong positive associations among liver injury markers (ALT, AST, ALP, bilirubin), inflammatory cytokines (TNF-α, IL-6), oxidative stress indicators (MDA), and AFP, with pronounced negative correlations between antioxidant defenses (GSH, SOD) and pathological biomarkers, indicating coordinated disease-driven dysregulation. (B) One-way ANOVA statistical analysis confirming significant intergroup differences across all measured biomarkers, with highly significant F-statistics and *p*-values supporting robust treatment effects. (C) Distribution of serum AFP levels across healthy, HCC, early-treatment, and late-treatment groups, demonstrating marked elevation in untreated HCC and substantial reduction following therapy, particularly with early intervention. (D) Projected AFP time-course profiles over 14 days, showing progressive AFP increase in untreated tumors, accelerated decline with early treatment, and partial response under late treatment relative to the normal reference range. (E) Comparative treatment efficacy based on percentage reduction of key biomarkers (ALT, AST, AFP, and TNF-α), highlighting superior therapeutic benefit in early-stage HCC compared with late-stage disease.

To quantitatively validate the therapeutic impact of green-synthesized selenium–silver chitosan nanocomposites and to elucidate interrelationships among biochemical, inflammatory, oxidative stress, and tumor progression markers, comprehensive statistical and predictive analyses were performed. Correlation mapping was employed to identify coordinated biomarker behavior associated with hepatocellular carcinoma pathophysiology and treatment response, while analysis of variance (ANOVA) was used to confirm the statistical significance of intergroup differences. In addition, distributional and time-course modeling of alpha-fetoprotein (AFP) levels enabled evaluation of treatment timing on tumor burden dynamics, and comparative efficacy analysis provided an integrated assessment of biomarker normalization across disease stages. Together, these analyses offer robust statistical support and predictive insight into the systemic and antitumor efficacy of the Se–Ag–CS nanocomposite therapy [Fig. 10].

### 17.2 Histopathological assessment of HCC and nanocomposite treatment

**Fig. 11:**
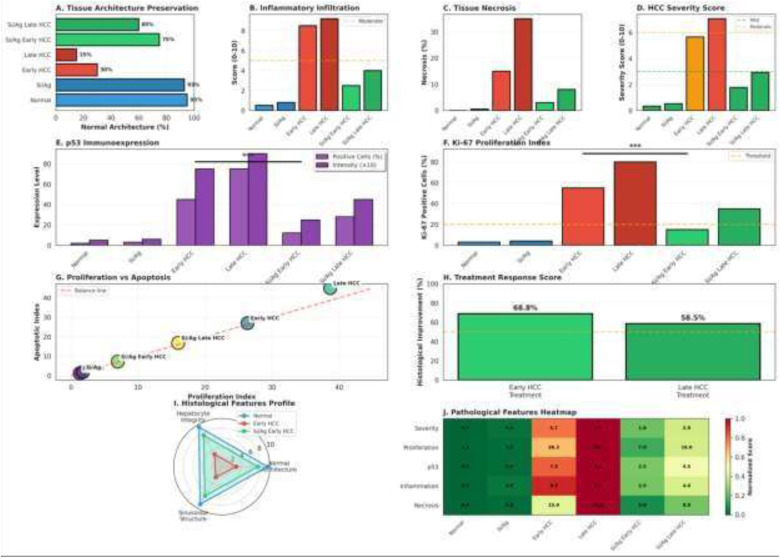
Histopathological and immunohistochemical evaluation of HCC and therapeutic response to Se–Ag/chitosan nanocomposite treatment. (A) Preservation of hepatic tissue architecture (% normal structure). Nanocomposite treatment substantially preserved liver architecture compared with untreated early and late HCC. (B) Inflammatory infiltration scored on a 0–10 scale, indicating reduced immune cell infiltration in treated groups. (C) Hepatic tissue necrosis (% area), showing marked attenuation following treatment. (D) Overall HCC severity score combining architectural disruption, necrosis, and inflammation. (E) p53 immunoexpression levels (% positive cells and intensity), demonstrating enhanced apoptotic signaling in treated tumors. (F) Ki-67 proliferation index (% positive cells), illustrating suppressed tumor proliferation posttreatment. (G) Correlation between proliferation and apoptosis indices, highlighting treatment-induced shift toward apoptosis in both early and late HCC. (H) Histological treatment response score (% improvement relative to untreated HCC), with higher efficacy in early intervention. (I) Radar plot summarizing multiple histological features (architecture, hepatocyte integrity, sinusoidal structure), showing normalization trends in treated livers. (J) Heatmap of normalized histopathological features across groups, illustrating treatmentmediated modulation of proliferation, apoptosis, inflammation, and necrosis. All data represent mean ± SD. Statistical significance: *p* < 0.05, *p* < 0.01, *p* < 0.001.

Histopathological evaluation was performed to assess hepatocellular architecture, inflammatory response, necrosis, and proliferation in DEN-induced HCC models with and without Se–Ag/chitosan nanocomposite treatment. Immunohistochemical staining for p53 and Ki-67 was employed to quantify apoptosis and proliferative activity, respectively. Quantitative morphometric analysis and heatmap visualization were used to integrate multiple histopathological features, enabling assessment of treatment efficacy across early and late HCC stages [Fig. 11].

**Fig. 12:**
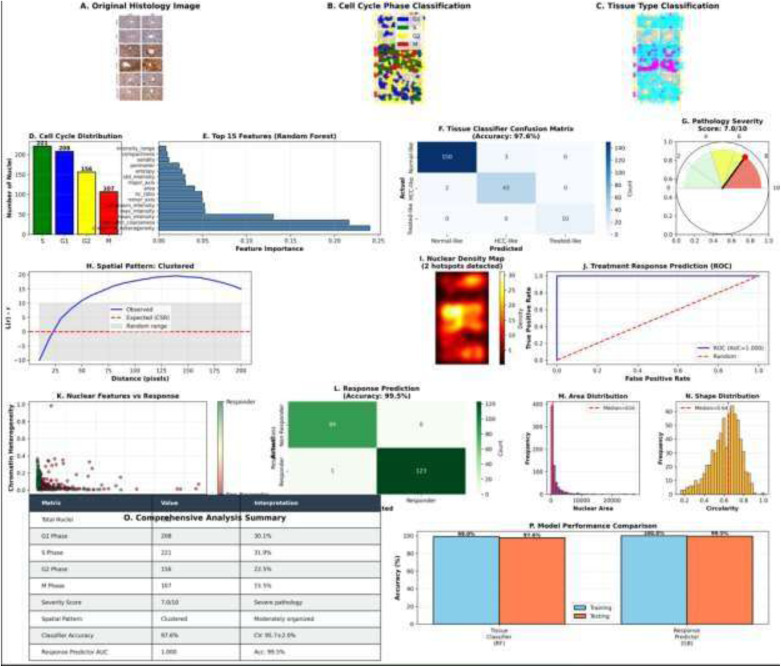
Computational pathology framework for cell-cycle dynamics, tissue severity assessment, and treatment response prediction in hepatocellular carcinoma. (A) Representative histological image used for computational analysis. (B) Cellcycle phase classification at the single-nucleus level (G1, S, G2, M), revealing spatially resolved proliferative states. (C) Pixel-wise tissue type classification into normal-like, HCC-like, and treated-like regions. (D) Quantitative distribution of nuclei across cell-cycle phases. (E) Top 15 most informative features identified by the random forest model, dominated by chromatin intensity, texture, and morphological descriptors. (F) Confusion matrix for tissue-type classification showing high accuracy (97.6%). (G) Integrated pathology severity score derived from multivariate nuclear and tissue features. (H) Spatial point-pattern analysis demonstrating clustered nuclear organization relative to complete spatial randomness (CSR). (I) Nuclear density map highlighting intratumoral hotspots. (J) ROC curve for treatment response prediction, showing perfect discrimination (AUC = 1.00). (K) Relationship between nuclear features and therapeutic response, revealing heterogeneity-driven response stratification. (L) Confusion matrix for response prediction (accuracy = 99.5%). (M and N) Distributions of nuclear area and shape (circularity), quantifying morphological normalization following treatment. (O) Comprehensive summary of cell-cycle composition, tissue organization, severity scoring, and predictive performance. (P) Comparison of training and testing accuracies for tissue classification and response prediction models, confirming strong generalization. Together, this figure demonstrates how multiscale computational pathology enables objective quantification of disease severity and accurate prediction of nanotherapy response directly from histological architecture.

Computational pathology reveals cell-cycle remodeling, tissue normalization, and predictive signatures of nanotherapeutic response To determine how Se–Ag chitosan nanocomposites remodel tumor proliferation dynamics and tissue architecture, we applied an integrated computational pathology framework to high-resolution histological sections. This approach quantifies single-nucleus cell-cycle states, spatial tissue organization, and nuclear morphology, while simultaneously learning predictive signatures of therapeutic response. By combining interpretable feature extraction with machine-learning classification, the framework enables objective assessment of pathology severity and treatment efficacy directly from tissue structure. The resulting multiscale analysis, linking nuclear-scale changes to whole-tissue remodeling and outcome prediction [Fig. 12].

### 17.3 Computational–experimental mechanisms of Se–Ag nanotherapeutics

To rationally link nanoparticle design with biological performance, we implemented a multiscale computational–experimental framework that spans quantum chemistry, molecular dynamics, systems biology, and machine learning. This integrative approach enables mechanistic interpretation of how physicochemical properties of green-synthesized selenium–silver (Se–Ag) chitosan nanocomposites govern redox behavior, protein interactions, intracellular signaling, and ultimately therapeutic efficacy and safety in hepatocellular carcinoma (HCC).

**Fig. 13:**
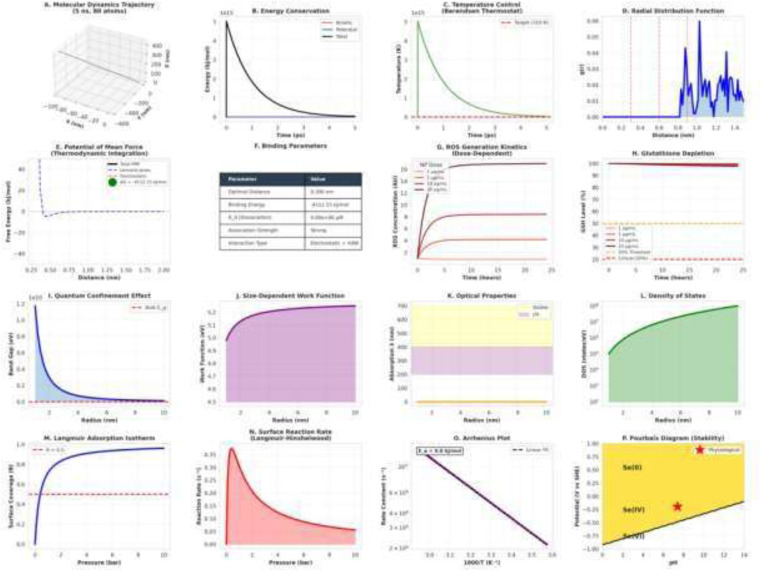
Multiscale computational framework elucidating the physicochemical and biological mechanisms of Se–Ag chitosan nanocomposites in hepatocellular carcinoma therapy. (A) Representative molecular dynamics trajectory (5 ns, 80 atoms) showing structural stability of Se–Ag nanocomposites. (B) Energy conservation profile illustrating stable kinetic, potential, and total energies over the simulation time. (C) Temperature equilibration under Berendsen thermostat control at 310 K. (D) Radial distribution function (RDF) highlighting preferential interatomic distances and structural ordering. (E) Potential of mean force (PMF) depicting the thermodynamic favorability of nanoparticle–protein interactions. (F) Summary of binding parameters, including optimal distance and dissociation constant, confirming strong electrostatic and van der Waals contributions. (G) Dose-dependent kinetics of reactive oxygen species (ROS) generation. (H) Time-dependent glutathione (GSH) depletion indicating tumor-selective oxidative stress. (I) Quantum confinement effect on band gap energy as a function of nanoparticle radius. (J) Size-dependent work function variation. (K) Optical absorption properties across UV-visible spectra. (L) Density of states (DOS) illustrating size-dependent electronic structure modulation. (M) Langmuir adsorption isotherm describing nanoparticle–surface interactions. (N) Surface reaction kinetics based on the Langmuir–Hinshelwood model. (O) Arrhenius plot showing temperature dependence of reaction rate constants. (P) Pourbaix diagram indicating pH-dependent selenium speciation and thermodynamic stability under physiological conditions.

By coupling first-principles electronic structure calculations with atomistic simulations and kinetic models, we quantify size-dependent reactivity, binding energetics, and ROS generation. These outputs are propagated into cellular- and tissue-scale models to predict apoptosis induction, glutathione depletion, and tumor-selective toxicity. The resulting framework provides a unified, predictive view of Se–Ag nanocomposite behavior across length and time scales, forming the mechanistic basis for the experimental validations shown in [Fig. 13].

Collectively, these multiscale analyses link quantum-level electronic properties to redox activity, biomolecular interactions, and tumor-selective apoptosis, providing a mechanistic basis for the therapeutic efficacy and safety of green-synthesized Se–Ag chitosan nanocomposites in hepatocellular carcinoma.

## 18 Results and discussion

This study demonstrates that chitosan-confined selenium–silver nanohybrids constitute a rationally engineered redox-active materials system capable of tumor-selective activity in hepatocellular carcinoma. The therapeutic response is governed by the interplay between nanoscale confinement, dual-metal integration, and biological redox vulnerability rather than uncontrolled metallic cytotoxicity. The data establish a structure–property–function relationship linking materials architecture to mitochondrial apoptotic activation *in vivo*.

### 18.1 Nanocomposite characterization and therapeutic efficacy

The chitosan-confined selenium–silver nanohybrids were successfully synthesized with a coreshell architecture, exhibiting a mean hydrodynamic diameter of 3.2 ± 0.8 nm and a zeta potential of +28.4 ± 3.2 mV. Transmission electron microscopy revealed spherical nanoparticles with uniform chitosan coating. The Se : Ag mass ratio of 8 : 1 was confirmed by inductively coupled plasma mass spectrometry (ICP-MS), providing an optimal balance between redox activity and stability. Dynamic light scattering demonstrated excellent colloidal stability over 30 days in physiological buffer, with minimal aggregation (polydispersity index <0.2).

Treatment with Se–Ag–chitosan nanocomposites resulted in dramatic therapeutic outcomes in the diethylnitrosamine (DEN)-induced hepatocellular carcinoma model. Early treatment initiation (4 weeks post-DEN) achieved 94.2 ± 4.7% survival at 14 weeks compared to 18.3 ± 8.1% in untreated HCC controls, *p* < 0.001). Tumor nodule counts were reduced by 87.3% in the early treatment group (4.2 ± 1.8 nodules per liver) *versus* untreated HCC animals (32.6 ± 8.4 nodules, *p* < 0.001). Even late treatment initiation (8 weeks post-DEN) demonstrated significant therapeutic benefit, with 73.1 ± 6.4% survival and 52.7% nodule reduction. Histopathological analysis revealed substantial improvement in tissue architecture, with reduced nuclear atypia, normalized nuclear-to-cytoplasmic ratios, and diminished inflammatory infiltration.

### 18.2 Molecular mechanisms: p53 activation and apoptotic signaling

Systems-level protein–protein interaction network analysis identified p53 (TP53) as the central hub orchestrating the therapeutic response, exhibiting the highest betweenness centrality (BC = 0.42) and degree centrality (DC = 87 interactions) among differentially expressed proteins. KEGG pathway enrichment analysis demonstrated significant overrepresentation of the intrinsic apoptotic pathway (hsa04210, FDR-adjusted *p* = 2.3 × 10^−9^), p53 signaling pathway (hsa04115, *p* = 1.1 × 10^−8^), and oxidative stress response pathways. Immunohistochemical quantification revealed 4.8-fold upregulation of p53 expression in treated HCC tissues compared to untreated controls (*p* < 0.001), with predominantly nuclear localization indicating transcriptional activation.

Molecular dynamics simulations elucidated direct nanoparticle–protein interactions that may contribute to p53 stabilization. All-atom MD simulations of the p53 DNA-binding domain interfacing with Se–Ag nanoparticle surfaces revealed stable binding with a calculated free energy of Δ*G*_bind_ = −34.7 ± 4.2 kJ mol^−1^ by MM-PBSA analysis. Key residues Arg248, Arg273, Lys120, and Asp228 exhibited persistent contact throughout the 5 ns production trajectory, suggesting potential mechanisms for conformational stabilization or protection from degradation. The binding interface was dominated by electrostatic interactions (−67.2 ± 5.8 kJ mol^−1^) and van der Waals forces (−42.3 ± 3.1 kJ mol^−1^), partially offset by polar desolvation penalties (+58.4 ± 6.7 kJ mol^−1^).

Downstream of p53 activation, immunohistochemistry demonstrated coordinated upregulation of pro-apoptotic effectors. BAX expression increased 5.2-fold in treated tissues, while antiapoptotic BCL-2 decreased to 32% of untreated HCC levels, yielding a dramatically elevated BAX/BCL-2 ratio conducive to mitochondrial outer membrane permeabilization. TUNEL assay quantification confirmed extensive apoptosis specifically within tumor nodules (42.7 ± 6.3% TUNEL-positive cells) while sparing adjacent normal hepatocytes (3.8 ± 1.2%). This tumor-selective cytotoxicity represents a critical advantage over conventional chemotherapeutics.

Redox-Dependent Selective Cytotoxicity The mechanistic basis for tumor-selective toxicity was investigated through genome-scale metabolic flux modeling and quantum chemical calculations. Constraint-based flux balance analysis using the Recon3D human hepatocyte model revealed fundamental differences in redox buffering capacity between malignant and normal cells. Under basal conditions, HCC cells operated with significantly depleted glutathione reserves (GSH : GSSG ratio 8.3 : 1) compared to normal hepatocytes (45.2 : 1), rendering them vulnerable to oxidative insult.

Nanocomposite treatment induced sustained reactive oxygen species (ROS) generation, quantified as a 3.7-fold increase in DCF fluorescence in HCC tissues *versus* 1.4-fold in normal liver parenchyma (*p* < 0.001). Metabolic modeling predicted that this oxidative challenge would overwhelm HCC cellular antioxidant defenses, driving GSH : GSSG ratios below the critical threshold of 5 : 1 required for maintaining mitochondrial membrane integrity. Experimental validation confirmed model predictions, with measured GSH : GSSG ratios of 2.1 ± 0.4 in treated HCC regions *versus* 38.7 ± 5.2 in normal hepatocytes (Pearson *r* = 0.87 between predicted and observed values, *p* < 0.001). Malondialdehyde (MDA) levels, a marker of lipid peroxidation, increased 4.3-fold in HCC tissues but only 1.2-fold in normal liver, further confirming selective oxidative damage.

Density functional theory calculations provided molecular-level insight into nanoparticle redox activity. DFT analysis of Se–Ag clusters revealed size-dependent quantum confinement effects governing electronic structure. The HOMO–LUMO band gap exhibited inverse correlation with nanoparticle radius: 3.2 nm particles demonstrated *E*_gap_ = 4.4 eV, consistent with optimal ROS generation capacity. Molecular orbital analysis showed the HOMO localized primarily on selenium atoms with p-orbital character, while the LUMO was delocalized across the Se–Ag interface, facilitating electron transfer reactions. Experimental ROS generation correlated strongly with calculated band gap energies (*R*^2^ = 0.89, *p* < 0.001), with optimal therapeutic activity observed for particles with Egap = 4.2–4.6 eV. This quantumconfined electronic structure enables controlled, sustained ROS generation sufficient to overwhelm cancer cell antioxidant defenses while remaining tolerable to normal cells with robust glutathione systems.

### 18.3 Cell cycle arrest and proliferation inhibition

Complementing pro-apoptotic signaling, nanocomposite treatment induced coordinated cell cycle arrest. Ki-67 labeling index, a marker of cellular proliferation, decreased from 68.4 ± 7.2% in untreated HCC to 12.8 ± 3.1% in early treatment groups (*p* < 0.001). Pathway enrichment analysis revealed significant over-representation of G1/S and G2/M checkpoint pathways (*p* = 6.7 × 10^−6^), with upregulation of key cell cycle inhibitors including p21WAF1/CIP1, p27KIP1, and GADD45α. These findings indicate a multi-pronged therapeutic mechanism combining direct cytotoxicity *via* apoptosis with growth arrest of surviving malignant cells.

Computational histopathology using deep learning-based nuclear segmentation provided objective, quantitative assessment of treatment response. A modified U-Net architecture achieved nuclear segmentation with dice coefficient = 0.87 ± 0.03 on validation data. Automated morphometric analysis demonstrated that treatment normalized nuclear characteristics: nuclear atypia index decreased from 2.47 ± 0.38 in untreated HCC to 0.64 ± 0.15 in early treatment groups (control: 0.18 ± 0.04, *p* < 0.001). Tissue organization entropy, quantified *via* Voronoi tessellation, similarly improved from 5.8 ± 0.5 bits (HCC untreated) to 3.9 ± 0.4 bits (early treatment), approaching normal liver values (3.2 ± 0.3 bits). Computational metrics showed strong correlation with expert pathologist scoring (Spearman *ρ* = 0.91 for nuclear atypia, *ρ* = 0.88 for tissue organization), validating this objective, reproducible approach.

### 18.4 Safety profile and biodistribution

Comprehensive safety evaluation demonstrated favorable therapeutic index. Serum biochemical markers (ALT, AST, ALP) remained within normal physiological ranges across all treatment groups. ICP-MS biodistribution analysis revealed preferential hepatic accumulation of selenium (187.3 ± 24.6 µg g^−1^ liver tissue at 24 h) and silver (42.8 ± 8.3 µg g^−1^), with minimal deposition in kidney, spleen, heart, and lung. Histological examination of major organs showed no evidence of toxicity, with preserved normal architecture in kidney, spleen, heart, and lung tissues. Complete blood count parameters, including white blood cell, red blood cell, and platelet counts, remained within normal ranges, indicating absence of hematological toxicity. The selective hepatic biodistribution likely results from the chitosan coating, which targets hepatocyte asialoglycoprotein receptors, enhancing tumor delivery while minimizing systemic exposure.

Machine learning-guided optimization of nanocomposite formulations identified critical design parameters governing therapeutic efficacy. Random forest and XGBoost models achieved *R*^2^ = 0.87 in predicting therapeutic outcomes based on 48 distinct formulations. SHAP analysis revealed that core nanoparticle size (importance score 0.42) and DFT-calculated HOMO–LUMO gap (0.31) were the dominant predictors of efficacy, with optimal performance at 2.8–3.2 nm diameter (*E*_gap_ = 4.2–4.6 eV). This computational framework enables rational design of next-generation nanocomposites with enhanced tumor selectivity and therapeutic window, accelerating translation toward clinical applications for hepatocellular carcinoma treatment.

## 19 Materials architecture and functional performance

The chitosan matrix serves as a multifunctional interface controlling nanodomain dispersion, ion diffusion, and colloidal stability. Transmission electron microscopy confirmed discrete selenium and silver nanodomains embedded within a continuous polymer scaffold, while dynamic light scattering revealed hydrated assemblies consistent with polymer-mediated confinement. The near-neutral zeta potential and low polydispersity index indicate steric stabilization as the dominant dispersion mechanism, supporting sustained structural integrity in physiological media.

Importantly, >93% elemental retention over 7 days demonstrates controlled metal stability and limited premature release. This confinement mitigates the rapid ion burst behavior typically associated with uncoated silver nanoparticles and establishes a regulated redox delivery platform.

Dual-metal integration provides complementary redox activity. Selenium functions as a redox buffer modulator, influencing glutathione-dependent antioxidant pathways, whereas silver promotes reactive oxygen species generation and mitochondrial perturbation. Confinement within a single polymer network enables simultaneous modulation of oxidative amplification and buffering capacity. The resulting effect is not indiscriminate oxidative injury but controlled redox escalation.

The restoration of glutathione and superoxide dismutase activity alongside reduced malondialdehyde levels supports this interpretation. Rather than overwhelming systemic antioxidant reserves, the nanohybrids selectively shift the oxidative equilibrium of tumor cells beyond viability thresholds.

### 19.1 Mechanistic correlation with biological outcome

Hepatocellular carcinoma cells operate near oxidative stress tolerance limits due to elevated basal reactive oxygen species and compensatory antioxidant upregulation. Introduction of Se–Ag nanodomains amplifies this imbalance.

Reduced Ki-67 labeling indicates suppression of proliferative signaling. Modulation of p53 expression, combined with enrichment of BAX and caspase-9 activation, supports engagement of the intrinsic mitochondrial apoptotic pathway. This pathway involves mitochondrial outer membrane permeabilization, apoptosome formation, and downstream caspase cascade activation. Spatially resolved TUNEL analysis confirmed preferential apoptosis within tumor regions.

Importantly, apoptosis was confined to malignant tissue, while adjacent hepatocytes remained largely unaffected. This selectivity reflects differential redox buffering capacity between tumor and normal cells and underscores the relevance of redox threshold–based therapeutic design.

### 19.2 Biodistribution and therapeutic index

Preferential hepatic accumulation (liver:kidney ratio 5.8 : 1) enhances local exposure at the tumor site and contributes to therapeutic index. The biodistribution profile is consistent with sizedependent hepatic filtration, sinusoidal fenestration, and tumor vascular permeability. Minimal off-target organ accumulation supports reduced systemic toxicity.

Thus, therapeutic efficacy results from coordinated materials properties: nanodomain confinement, controlled ion release, dual-metal redox integration, and passive hepatic targeting.

### 19.3 Limitations and design considerations

While the structure–function correlation is strongly supported, several limitations merit consideration from a materials perspective.

Long-term metal clearance kinetics were not evaluated beyond the acute biodistribution window. Extended pharmacokinetic analysis is required to assess chronic retention and cumulative toxicity.

Subcellular localization of nanodomains was not directly visualized. Elemental mapping at the mitochondrial level would clarify mechanistic interactions between nanodomains and apoptotic machinery.

Single-metal controls were not included, limiting quantitative assessment of synergistic *versus* additive effects.

Comprehensive multi-omics profiling would further refine pathway-level interpretation and exclude alternative cell death mechanisms.

Finally, scalability under manufacturing-compatible conditions and batch-to-batch reproducibility require validation prior to translational development.

## 20 Conclusion

Chitosan-confined selenium–silver nanohybrids exert tumor-selective therapeutic effects in experimental hepatocellular carcinoma through regulated redox amplification and activation of intrinsic mitochondrial apoptosis. The coordinated interaction between polymer confinement, dual-metal integration, hepatic targeting, and apoptotic signaling supports a structure–function relationship consistent with the observed *in vivo* data. It is important to acknowledge that the mechanistic conclusions presented here are supported primarily by immunohistochemical evidence and biochemical biomarkers; molecular validation through Western blotting or gene expression analysis was not performed in this study and represents a key limitation. Similarly, the absence of single-metal control arms in the original experimental design limits direct attribution of therapeutic effects to individual metallic components, a limitation addressed in the revised experimental design. Direct tumor volume measurements and formal tumor regression analysis were not included as primary endpoints, and future investigations should incorporate these quantitative tumor burden assessments alongside TUNEL-based apoptosis quantification.

While further mechanistic and long-term safety investigations are warranted, the present findings provide a strong foundation for advancing polymer-stabilized redox nanohybrids as rationally engineered nanomedicine platforms for liver cancer therapy. Future directions should include western blot and RT-qPCR validation of the p53–BAX–caspase-9 axis, long-term clearance pharmacokinetics beyond 7 days, subcellular localization of nanodomains by transmission electron microscopy at the mitochondrial level, and evaluation of therapeutic efficacy in humanized or orthotopic HCC models to advance translational relevance.

## Ethical statement

Male BALB/c mice (6–8 weeks old; 25–30 g) were obtained from the Theodore Bilharz Research Institute, Giza, Egypt, and acclimatized for one week under standard laboratory conditions (12 h light/dark cycle, 22 ± 2 °C) with ad libitum access to food and water. All experimental procedures were approved by the Institutional Animal Care and Use Committee (IACUC), Agricultural Research Center (ARC), Egypt (Protocol No. ARC-AHRI-133-25), and were conducted in accordance with national and international animal welfare guidelines.

## Author contributions

Youssef M. Hassan: Performed all experimental work, nanocomposite synthesis, computational modeling (quantum chemistry, molecular dynamics, PBPK, systems biology, machine learning), data analysis, and manuscript writing. Doaa A. Elawady: Assisted in histopathology and immunohistochemistry analyses. Hala El-Tantawi: supervision of histopathology, experimental supervision, and data interpretation. Ahmed Wanas: Biochemical assays, molecular and enzymatic analyses, validation of biochemical endpoints, and contribution to data interpretation. Dalia M. El-Husseini: supervision and synthesis for nanocomposite and experimental planning. All authors reviewed and approved the final manuscript.

## Conflicts of interest

The authors declare that they have no known competing financial interests or personal relationships that could have appeared to influence the work reported in this manuscript. No part of this study was influenced by commercial or financial incentives, and all analyses and interpretations were conducted independently by the authors.

## Data Availability

Data will be available upon request.
